# Assessing photodynamic therapy under in vitro hypoxia: photochemical pathways and methodological challenges

**DOI:** 10.1007/s12551-026-01457-4

**Published:** 2026-09-23

**Authors:** Ieda Vieira da Cunha, Mayara Martins Caetano, Naiara Cristina Bessas, Juliana Cristina Biazzotto, Maynne Duarte Suriani Franco, Laísa Bonafim Negri, Maria Vitória Pereira Vaz, Patrícia Alves de Matos, Renata Galvão de Lima, Tayana Mazin Tsubone

**Affiliations:** 1 Institute of Chemistry, Federal University of Uberlândia, Uberlândia, Minas Gerais Brazil; 2 Pharmaceutical Science Faculdade of Ribeirão Preto, University of São Paulo, Ribeirão Preto, São Paulo, Brazil; 3 Department of Medicine, Division of Infectious Diseases, Harvard Medical School, Boston, MA USA

**Keywords:** PDT, Hypoxia experimental protocol, ROS, Biomolecule oxidation

## Abstract

Photodynamic therapy (PDT) is a light-activated modality that kills cells through photosensitizer-triggered reactive oxygen species, but its efficacy is strongly shaped by oxygen availability and by whether photochemistry proceeds through Type I or Type II pathways. In oxygen-rich settings, Type II PDT mainly generates singlet oxygen, whereas hypoxia suppresses this route and shifts interest toward Type I mechanisms that produce radical species such as superoxide, hydroxyl radicals, and hydrogen peroxide. This review argues that hypoxia-driven resistance in vitro is not explained by oxygen depletion alone, but by interactions among photochemical pathway choice, cellular adaptation, photosensitizer uptake and localization, and assay limitations. It highlights that hypoxia can reshape membrane composition, vesicular trafficking, and organelle targeting, which in turn alters reactive oxygen species patterns and photodamage outcomes. Methodologically, the work recommends Controlled hypoxia models, including 2D low oxygen cultures and 3D spheroids, together with orthogonal readouts of hypoxia and oxidative damage. For ROS profiling, it supports comparing normoxic and hypoxic conditions using probes for total ROS, superoxide, hydroxyl radicals, and singlet oxygen, because no single assay captures the full mechanistic picture. Finally, it emphasizes that lipid and protein oxidation should be interpreted with caution in hypoxic PDT, since FOX, TBARS, iodometric methods, and BODIPY-C11 mainly report secondary or localized products and may miss Type I driven damage unless combined with imaging, membrane integrity assays, and time-resolved measurements.

## Introduction

**Fig. 1:**
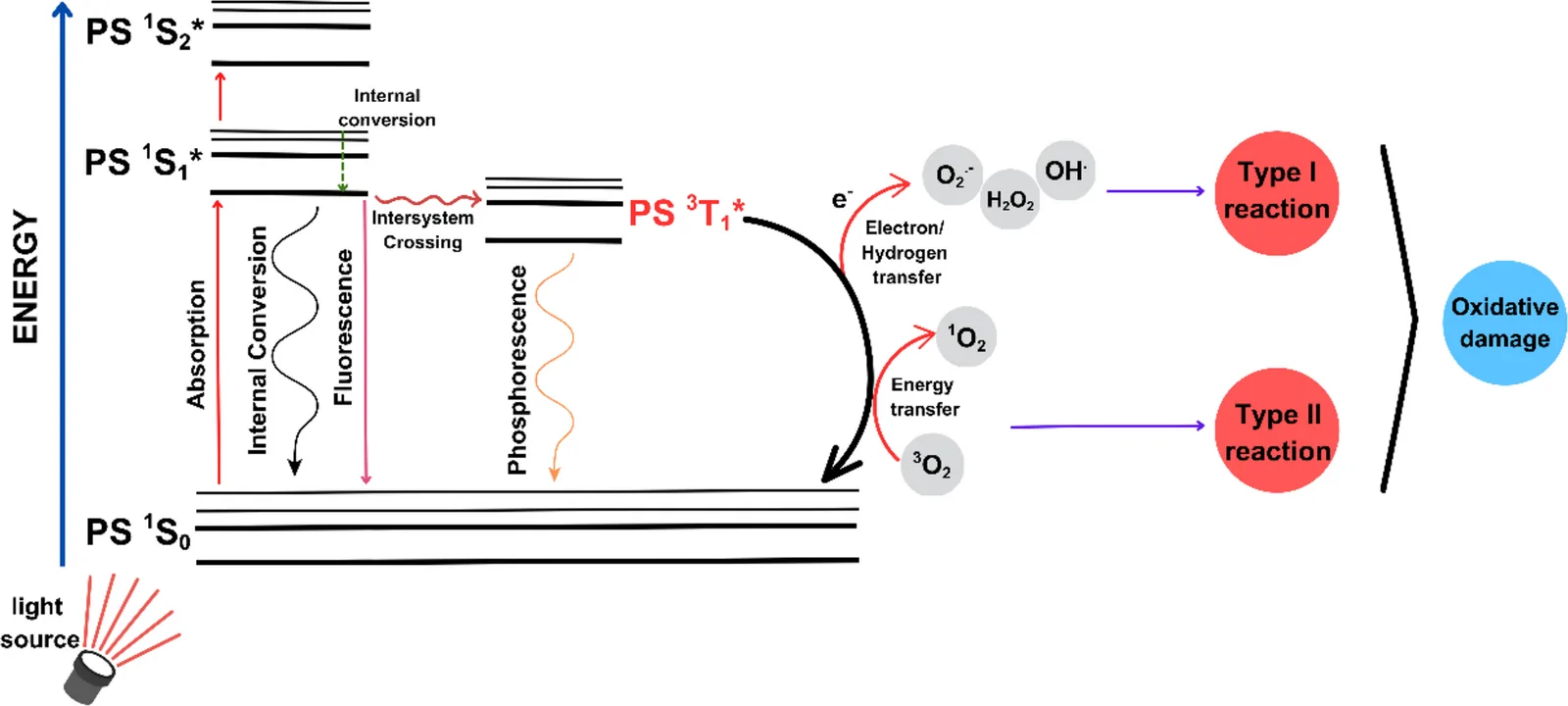
Representation of Jablonski diagram in PDT context

Photodynamic therapy (PDT) is a light-activated therapeutic modality that relies on the excitation of a photosensitizer (PS) to generate reactive oxygen species (ROS) capable of inducing oxidative damage and cell death. Upon photoexcitation, PSs populate excited singlet and triplet states, as described by the Jablonski diagram (Fig. 1) (Jabłoński 1935), enabling two major photochemical pathways: the Type II mechanism, based on energy transfer to molecular oxygen to produce singlet oxygen (^1^O_2_), and the Type I mechanism, which involves electron or hydrogen transfer reactions generating radical species such as superoxide (O_2_^•^⁻), hydroxyl radicals (^•^OH), and hydrogen peroxide (H_2_O_2_) (Baptista et al. 2017).

The production of ^1^O_2_ depends on triplet-triplet energy transfer (TETT) between triplet oxygen (^3^O_2_) and the PSs in triplet state excited (PS ^3^T_1_^*^) by intersystem crossing (ISC) as shown in Equations (1) and (2) (Wu et al. 2017). In the Type II mechanism, energy transfer from the PS in the triplet excited state to triplet oxygen results in the generation of singlet oxygen (^1^O_2_):

1 $$PS^1S_0\rightarrow PS^1S_1^\ast\rightarrow PS^3T_1^\ast$$

2 $$PS^3T_1^\ast+{}^30_2\rightarrow PS^1S_0+O_2$$

When the PS is in the excited triplet state (PS ^3^T_1_*), electron transfer can occur from PS to the suppressor Q (e.g., vitamin C, riboflavin, reduced coenzymes (Pereira et al. 2017), amino acids, and nitrogenous bases to be oxidized), producing a radical pair: PS ^3^T_1_^*^ + Q → PS^•−^ + Q^•+^. Starting from this radical pair, a cascade reaction of electron transfer can occur (Equations 3-5). The O_2_^•⁻^ radical anion can then be formed by electron transfer between the radical anion of PS and oxygen (PS^•−^ + O_2_ → PS ^1^S_0_ + O_2_^•⁻^). O_2_^•⁻^ is considered the primary reactive oxygen species generated through the Type I pathway and can subsequently give rise to hydroperoxyl radicals (HOO^•^), hydrogen peroxide (H_2_O_2_), and hydroxyl (^•^OH) through successive reactions (Equations 3–5) (Ochsner 1997; Da and Machado 2000).

3 $$O_2^{\bullet-}+PS^{\bullet-}+O_2\rightarrow HOO^\bullet+PS\left(-H\right)^\bullet$$

4 $$HOO^\bullet+PS\rightarrow H_2O_2+PS\left(-H\right)^\bullet$$

5 $$H_2O_2+O_2^{\bullet-}\rightarrow HO^\bullet+HO^-+O_2$$

O_2_^•−^ and H_2_O_2_ have limited biological toxicity that oppositely endows them broad diffusion range (O_2_^•−^ and H_2_O_2_ diffusion rate in H_2_O at 298 K 1.5 × 10^−8^ m^2^ s^−1^ and 2 × 10^−8^ m^2^ s^−1^, respectively) to generate the most reactive HO^•^ (diffusion rate = 3 × 10^−9^ m^2^ s^−1^ H_2_O at 298 K). ^•^OH is extremely destructive to almost all biological molecules (Li et al. 2022a), which enables the limited oxygen in the hypoxic tumor to be fully utilized. But it is still oxygen-dependent. The term “oxygen-independent” should not be used. As for Type II pathway, the PS ^3^T_1_^*^ transfers its energy to surrounding oxygen, forming the ^1^O_2_ with the decent cytotoxicity but short action range.

Traditionally, PDT has been conceptualized as a predominantly Type II process (Teng et al. 2025), implicitly assuming sufficient oxygen availability for efficient ^1^O_2_ generation. However, this paradigm is increasingly challenged by the recognition that oxygen availability is highly limited in solid tumor microenvironments, and both pre-existing hypoxia and additional oxygen consumption during illumination severely compromise ^1^O_2_ generation, making oxygen dependence a central cause of PDT failure in solid tumors (Lv et al. 2018; Li et al. 2022b).

In response to this limitation, recent studies have focused from purely ^1^O_2_‑centric designs toward PSs and photochemical schemes that either economize oxygen use or operate efficiently at low to negligible O_2_ levels (Li et al. 2020, 2022b). Comprehensive analyses of Type I PDT now show that appropriately engineered PSs can favor charge-separated states and electron transfer over energy transfer (Teng et al. 2025), thereby amplifying radical ROS such as O_2_^•^⁻ and ^•^OH and maintaining photocytotoxicity under hypoxic conditions where classical Type II agents fail (Yu et al. 2022). At the same time, hybrid Type I/II systems capable of generating both ^1^O_2_ and O_2_^•^⁻, such as boron-dipyrromethanes (BODIPYs) (Wang et al. 2012), naphthalimide derivatives (Nguyen et al. 2019), biotinylated porphyrinoids (An et al. 2022), and the methylene blue derivatives ENBS-B (Li et al. 2018a), ENBOS (Li et al. 2019), and SORgenTAM (Li et al. 2020), have demonstrated that radical-mediated photochemistry can partially circumvent oxygen dependence, revitalizing PDT efficacy under hypoxic conditions, underscoring the potential of mixed photochemical regimes to overcome hypoxia‑induced resistance.

These advances have stimulated rational design principles for Type I and Type I/II photodynamic agents, including tuning redox potentials, managing intersystem crossing and charge-separation lifetimes, and exploiting aggregation-induced emission to stabilize long-lived radical intermediates (Li et al. 2018b; Chen et al. 2021b; Teng et al. 2025). Yet, PS photophysical characterization remains dominated by measurements performed in air-saturated solutions—molar absorptivity, triplet lifetimes, and ^1^O_2_ quantum yields (*Φ*_Δ_)—while oxygen-dependent radical yields and their evolution under hypoxia are rarely quantified in a systematic fashion. As a result, PSs optimized to maximize *Φ*_Δ_ in normoxic media often display disappointing performance in hypoxic cellular assays, whereas Type I–biased candidates show robust activity that is not predictable from conventional *Φ*_Δ_ alone (Li et al. 2018b; Teng et al. 2025). These findings have shifted attention toward rational PS design strategies that favor electron-transfer processes, redox cycling, and intracellular radical amplification rather than exclusive reliance on ^1^O_2_ generation (Li et al. 2018b; Teng et al. 2025).

Modeling hypoxia in vitro represents a critical step toward understanding PDT resistance at the cellular level. Hypoxia can be induced using physical approaches, such as hypoxic chambers or incubators with controlled oxygen atmospheres (typically 1–5% O_2_) (Matthiesen et al. 2021a; Yu et al. 2022). While conventional two-dimensional (2D) monolayer cultures remain the most widely used models in PDT research, they fail to reproduce oxygen gradients, diffusion limitations, and metabolic heterogeneity characteristic of tumors. Three-dimensional (3D) spheroids and multicellular aggregates provide more physiologically relevant hypoxic microenvironments, often exhibiting intrinsic resistance to PDT compared to 2D cultures (Kucinska et al. 2021). Furthermore, prolonged hypoxic conditioning can select resistant cell populations with altered redox balance, mitochondrial function, and stress-response pathways, making cell line choice and culture architecture decisive variables in vitro PDT studies (Ferguson et al. 2018; Shirmanova et al. 2024).

Hypoxic tumor environments not only limit oxygen availability for Type II photoprocesses but also remodel the cellular microenvironment in ways that affect PS uptake and subcellular localization (Xu et al. 2019; Sun et al. 2020). Under low oxygen tension, hypoxia-inducible factor 1 (HIF-1)–driven pathways reprogram cellular metabolism (Infantino et al. 2021), alter lipid composition of membrane (Botto et al. 2008; Seo et al. 2022), modulate vesicular trafficking (Muñiz-García et al. 2022), and change the expression of transporters and stress-response proteins (Parks et al. 2016), which in turn may modify PS internalization efficiency, intracellular trafficking, and retention compared with normoxic conditions. Recent studies further show that redirecting PS localization among mitochondria (Xu et al. 2019), lysosomes (Lv et al. 2016a), or endoplasmic reticulum (Li et al. 2022c) markedly reshapes the spatial and temporal patterns of ROS formation and photodynamic response under hypoxia conditions (Ihata et al. 2022; Alva et al. 2024).

At the molecular level, PDT resistance in hypoxic settings is linked both to shifts in the dominant ROS species (Chen et al. 2021b; Alva et al. 2024) and to an altered antioxidant defense capacity (Kilinç and Güney 2019; Shekhovtsov et al. 2024). Moreover, many classical assays of oxidative damage to proteins and lipids were originally validated under normoxic conditions and may underestimate or distort the extent and nature of injury under reduced O_2_ (Hong et al. 2022; Du et al. 2022; Li et al. 2023).

Taken together, these observations indicate that PDT resistance observed in vitro under hypoxia is not merely a direct consequence of reduced oxygen availability for ^1^O_2_ formation, but rather arises from a complex interplay between competing photochemical pathways, hypoxia-driven cellular adaptations, and methodological constraints inherent to the models and assays used to evaluate PDT efficacy.

This review examines hypoxia-induced resistance to PDT in vitro cell models, with a particular focus on how experimental design and methodological choices influence the interpretation of PDT efficacy. By integrating these perspectives, the aim is to provide an experimental roadmap that helps in selecting and benchmarking next‑generation PSs in hypoxic tumor settings highlighting common pitfalls in current evaluation pipelines. Such an approach is essential for understanding and guiding the rational development of new PS.

## In vitro hypoxia methods

In vitro hypoxia models are essential to dissect how oxygen availability and microenvironmental architecture shape PDT response (Kucinska et al. 2021), especially when comparing Type II versus Type I/II PSs. Conceptually, these models can be grouped into the commonly 2D cell culture, where oxygen concentration is externally defined, and 3D platforms that generate intrinsic oxygen gradients and diffusion barriers closer to solid tumors (Wulftange et al. 2019; Salyha and Oliynyk 2023a).

### 2D monolayer

**Fig. 2:**
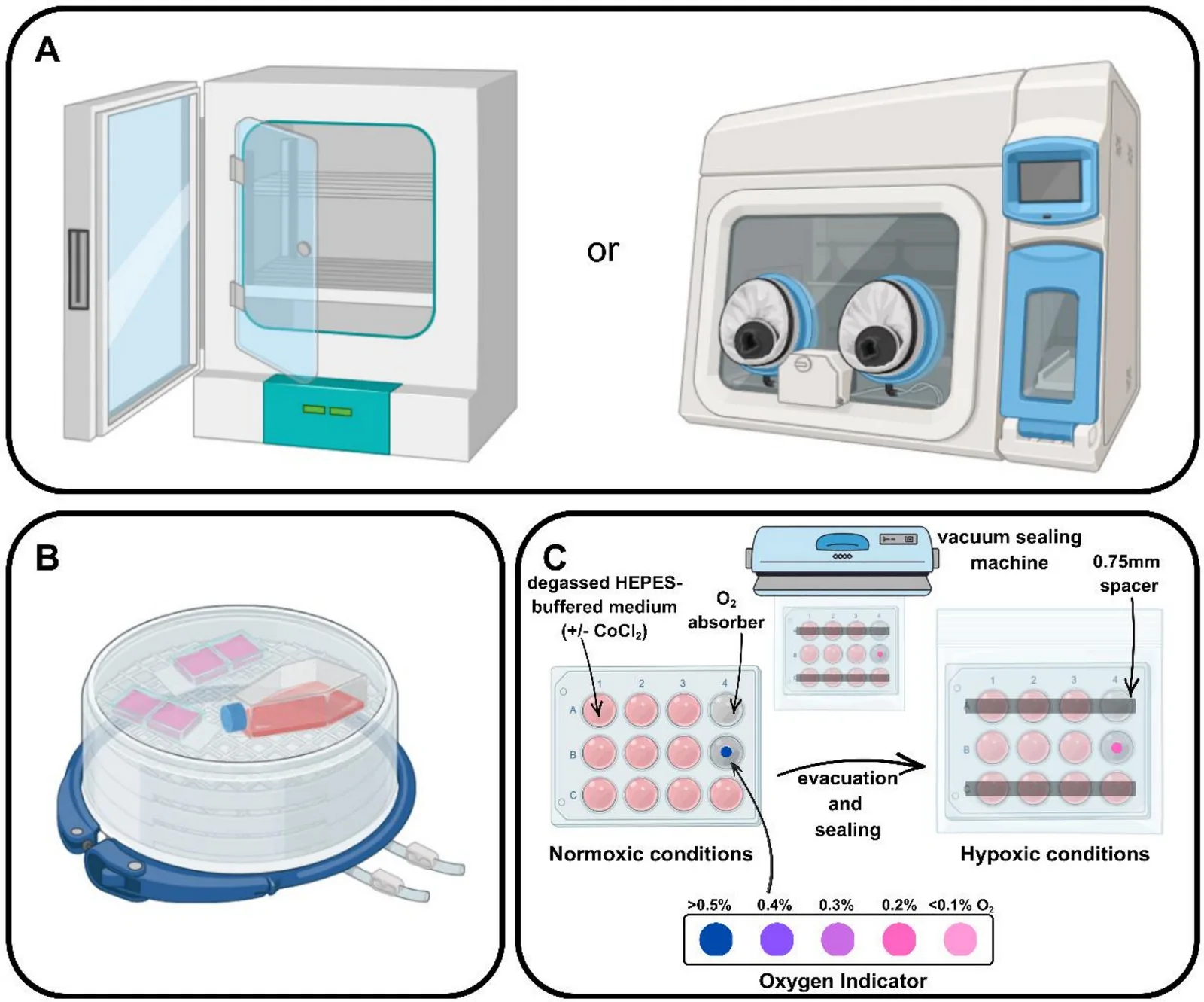
Experimental approaches used to establish hypoxic conditions in 2D cell culture models for PDT studies: (**A**) gas-controlled hypoxia incubators or workstations, (**B**) modular hypoxia chamber connected to premixed gas cylinders, and (**C**) vacuum-sealed hypoxia system using low-permeability bags and oxygen indicators. These methods are commonly used to maintain cells under low O_2_ conditions prior to PDT irradiation

In 2D monolayers cell culture, hypoxia is most commonly achieved using gas-controlled incubators (Fig. 2A) or modular chambers that maintain cells at 1–5% O_2_ for 24–48 h before PDT, yielding reproducible stabilization of HIF-1α and metabolic adaptation (Wu and Yotnda 2011). Commercial modular incubator chambers (Fig. 2B), such as polycarbonate units connected to premixed gas cylinders (Kaur et al. 2023), allow precise adjustment of O_2_, CO_2_, and humidity and can be placed inside standard CO_2_ incubators, providing a cost-effective alternative to dedicated hypoxia workstations for PDT studies. In addition, “vacuum bag” techniques (Fig. 2C), where culture vessels are sealed in low-permeability storage bags, evacuated, and backfilled with defined gas mixtures, offer a simple way to generate low oxygen environments without specialized incubators and have been successfully used to expose cells and tissues to controlled hypoxic conditions (Bakmiwewa et al. 2015; Matthiesen et al. 2021b). Under these conditions, several studies have reported marked reductions in ^1^O_2_ production and PDT efficacy for classical Type II PSs, consistent with an oxygen-limited regime (Nguyen et al. 2021; Chen et al. 2021b; Li et al. 2022b; Teng et al. 2025).

Chemical hypoxia mimetics provide a complementary approach in conventional incubators by stabilizing HIF-1α or decreasing mitochondrial oxygen consumption. Cobalt chloride (CoCl_2_) in the 100–600 µM range (Wu and Yotnda 2011; Kaur et al. (2023) is widely used to mimic chronic hypoxia in tumor cell lines by inhibiting prolyl hydroxylases and promoting HIF-1α accumulation, whereas desferrioxamine (DFO) at ~ 100 µM chelates iron and indirectly suppresses oxidative metabolism (Guo et al. 2006; Rinderknecht et al. 2021). These treatments reproduce key transcriptional hallmarks of hypoxia and are useful for probing hypoxia-driven resistance pathways; however, they do not fully recapitulate diffusion-limited oxygen gradients and may introduce off-target toxicity that must be carefully controlled in PDT experiments.

Beyond controlling external oxygen tension, it is essential to verify that cells are genuinely experiencing hypoxia at the molecular level using orthogonal readouts. Stabilization and nuclear accumulation of HIF-1α are commonly assessed by Western blot or immunofluorescence after 12–24 h at 1–5% O_2_ or in the presence of hypoxia mimetics (Wu and Yotnda 2011; Matthiesen et al. 2021b; Rinderknecht et al. 2021), providing a first-line confirmation that hypoxia-responsive signaling is activated.

In addition to HIF-1α immunodetection, several complementary assays are available to confirm that cells are truly hypoxic rather than merely exposed to reduced ambient O_2_. Oxygen-sensitive fluorescent probes such as Image-iT™ Green Hypoxia Reagent become fluorescent under low intracellular O_2_ concentration and have been used to visualize and quantify hypoxia in live cultures (Matthiesen et al. 2021b; Rinderknecht et al. 2021). Luciferase-based hypoxia reporters, in which firefly luciferase activity reflects HIF-dependent transcription, allow quantitative assessment of hypoxia-responsive signaling in living cells and have also been applied to validate hypoxic culture protocols (Wu and Yotnda 2011). In parallel, adduct formation by the 2-nitroimidazole pimonidazole under low pO_2_ can be detected by immunostaining or flow cytometry and is widely used as an independent marker of chronic or severe hypoxia (Aguilera and Brekken 2014).

### 3D spheroids cell model

Studies involving cell culture are widely used for clinical trials to aid in the development of new diagnostic tools and drug discovery. Two-dimensional (2D) cell culture approaches are gradually being replaced by three-dimensional (3D) cell culture methods because they provide more accurate data regarding the cell growth scenario (Chandra and Singh 2017; Mohammad-Hadi et al. 2018; Becceneri et al. 2020, 2024; Kucinska et al. 2021). As an in vitro model, three-dimensional cell culture allows researchers to conduct more biologically relevant studies than two-dimensional cell cultures (Engrácia et al. 2023).

**Fig. 3:**
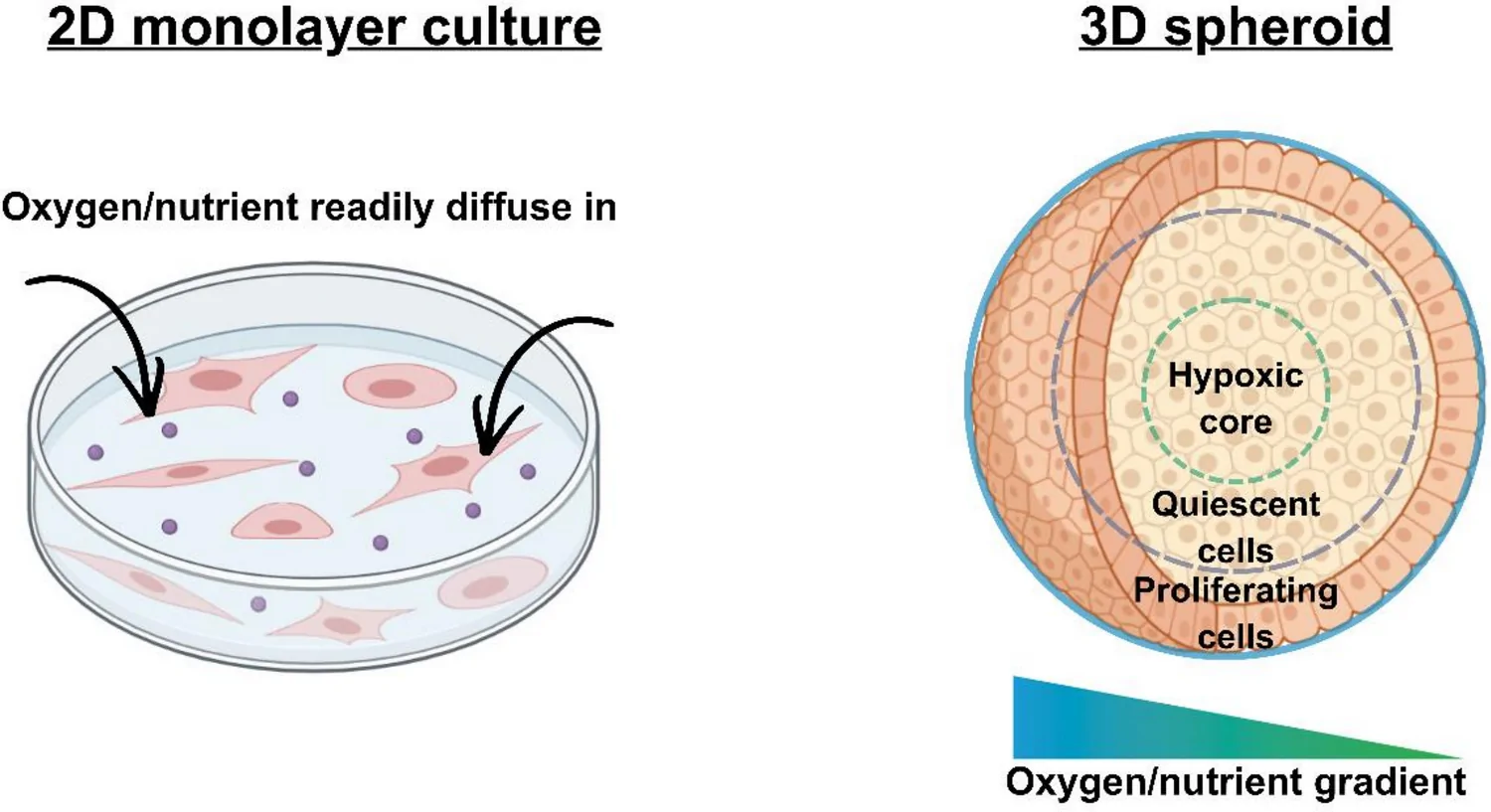
Schematic representation of conventional 2D monolayer and 3D spheroid culture models, illustrating oxygen and nutrient diffusion and cellular organization

In the 2D cell culture method (Fig. 3), the cell shape tends to be flat and elongated due to its ability to grow only on a two-dimensional surface, while in 3D cell culture (Fig. 3), the natural shape of the cell will be preserved during growth, allowing cell-to-cell interactions (Engrácia et al. 2023). In vivo, solid tumors are heterogeneously oxygenated and contain hypoxic regions that can lead to necrosis (Pereira et al. 2017). In order to mimic and better understand the action of photosensitizing drugs in tumor models using PDT, 3D spheroids have been used (Evans 2015; Pereira et al. 2017).

Spheroids can closely resemble the properties of tumors, as they are three-dimensional cell aggregates with a necrotic core surrounded by a layer of viable cells (Evans 2015; Pereira et al. 2017) and serve as excellent models for measuring the effects of oxygen diffusion and consumption, both during normal growth and PDT (Evans 2015).

In addition to the oxygen consumption in the architecture of 3D cell models, which mimic in vivo dynamics, and the penetration of visible light into 3D models, since the constructs are relatively thin (typically < 1 mm), partly to allow for oxygen replenishment, the attenuation of light for PDT activation becomes negligible (Mohammad-Hadi et al. 2018).

Some cell lines have the ability to produce spheroids, and other cell types will only produce cell aggregates. The cell heterogeneity and metabolic gradients (including oxygen, glucose, and ATP) become apparent in spheroids only when they are larger than 150 µm. Spheroids with a necrotic and hypoxic central region become apparent only at a diameter of ~ 500 µm. The necrotic central core model in 3D models occurs due to (i) nutrient and metabolite deficiency, (ii) variation in oxygen concentration, (iii) accumulation of waste products, and (iv) low pH. In in vivo solid tumor models, the necrotic core appears at a distance > 100 µm from a blood supply (Raza et al. 2017).

The spheroids’ size and hypoxic regions determine how they grow, behave, interact, and respond to the external environment, making it a crucial parameter for evaluating various properties. One of the challenges when working with 3D culture relates to image quality, where the images obtained must show accuracy in thickness and size laterally and perpendicularly. In cases of large spheroids, light transmission decreases (Yoo et al. 2024).

However, one of the challenges of working with 3D cells is visualizing cell growth and interactions compared to 2D models (Yu et al. 2022; An et al. 2022). Imaging techniques can provide valuable information about their dynamics and interactions with other cell types, helping to improve our understanding of tissue regeneration.

Specifically, studies involving PSs in 3D models are experimentally helpful since many PSs exhibit fluorescence emission and can be employed to dynamically visualize the uptake and localization of these agents at the cellular and subcellular level (Evans 2015). 3D spheroid cultures, which simulate avascular tumors, have been helpful in modeling the uptake and penetration of PSs through multiple cellular and subcellular levels.

Xiao et al. (2005), using a spheroid bladder cancer model with a diameter of less than 400 µm, examined the uptake distributions of several porphyrin PSs using confocal microscopy. The choice of different types of PSs was based on their lipophilic nature. Aluminum phthalocyanine chloride (AlPC) falls into the hydrophobic group. Protoporphyrin-IX (PpIX) and benzoporphyrin derivative monoacid ring A (BPD-MA) are on the borderline between the hydrophobic and amphiphilic groups. Hypocrellins (HBEA-R1) is amphiphilic. The results of the distribution of PSs in the spheroid showed that the hypocrellins and benzoporphyrin groups presented a greater intratumor distribution compared to AlPC and PpIX (Xiao et al. 2005).

In a recent study by Li et al*.* (2024), spheroids of MCF-7 human breast tumor cells were constructed and different doses of violet light at 408 nm and red light at 625 nm were applied under normal and hypoxic oxygen conditions in the presence of a PS. The cell viability results indicated that the light irradiated at 408 nm was more efficient at killing cells in the spheroids under normal oxygen conditions, while cells exposed to the hypoxic microenvironment exhibited minimal PDT-induced death (Li et al. 2024).

Thus, the systematic incorporation of spheroids into preclinical PDT studies represents a fundamental step toward increasing the translational relevance of experimental results. The continuous improvement of culture, imaging, and tumor microenvironment control techniques should further expand the potential of these models, contributing to the rational development of more effective PSs and optimized therapeutic protocols.

## Measuring ROS under normoxia and hypoxia conditions

ROS is a collective term for oxygen-derived molecules that are more reactive than molecular oxygen (O_2_), this group ^1^O_2_, O_2_⁻^•^, H_2_O_2_, ^•^OH, and HOO^•^ (Baptista et al. 2017). In PDT, the relative contribution of these species depends strongly on oxygen availability. Under hypoxia, ^1^O_2_ formation through classical Type II pathways decreases with falling pO2, whereas electron-transfer mediated Type I pathways may remain operative and become comparatively more relevant (Chen et al. 2021b; Teng et al. 2025). Therefore, comparing ROS generation under normoxic and hypoxic conditions may help distinguish whether a PS acts predominantly through Type II, Type I, or mixed photosensitizing mechanisms. The following subsections summarize experimental approaches used to detect ROS and evaluate oxidative processes in PDT systems.

### Detection of total ROS

**Fig. 4:**
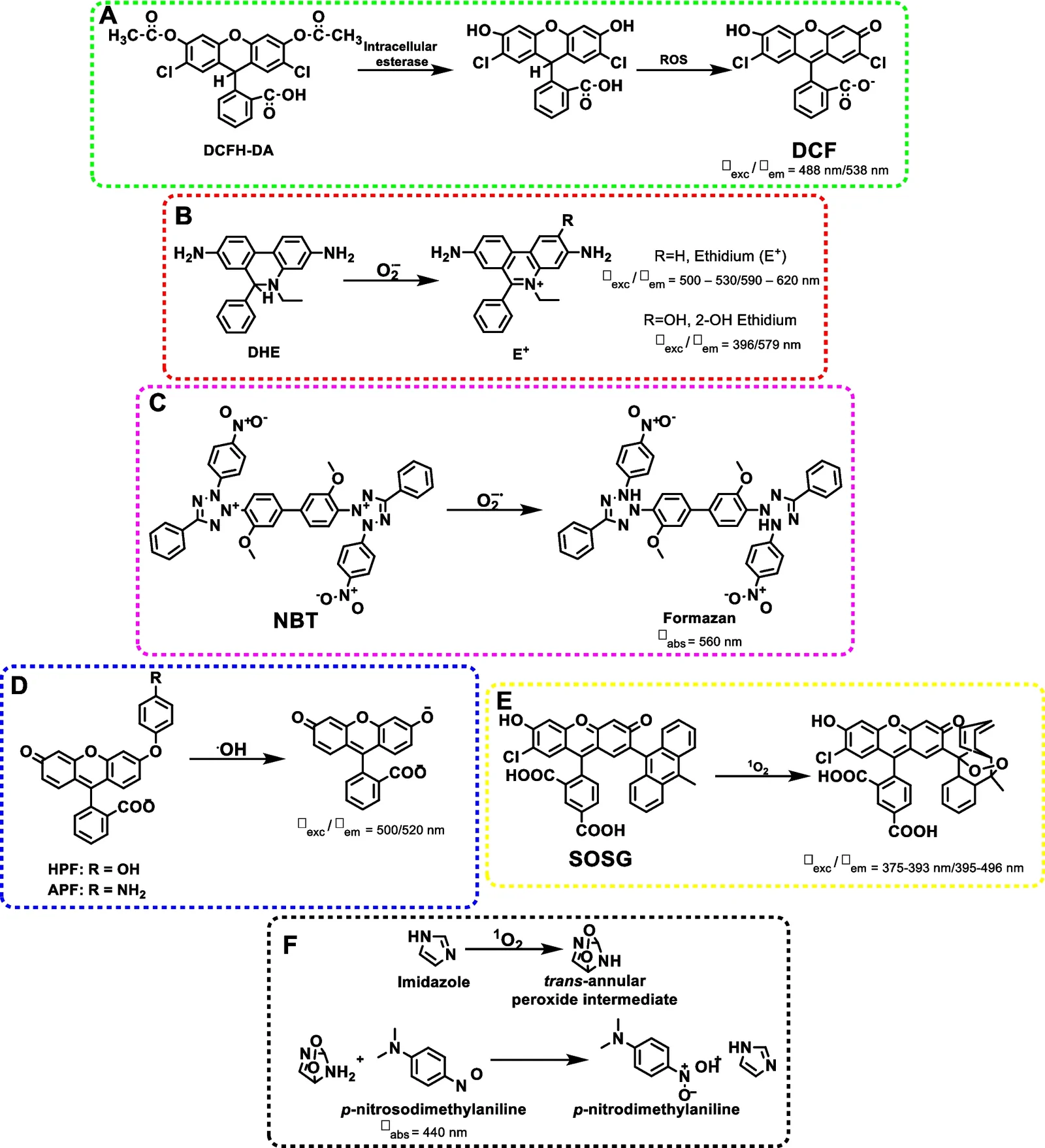
Schematic representation of the chemical mechanisms involved in the detection and quantification of reactive oxygen species (ROS) in vitro: (**A**) ROS detection using DCFH-DA; (**B**) superoxide anion (O_2_•⁻) detection using DHE; (**C**) superoxide anion (O_2_•⁻) detection using NBT; (**D**) hydroxyl radical (•OH) detection using APF and HPF fluorescent probes; (**E**) singlet oxygen (^1^O_2_) detection using the SOSG probe and **F** RNO assay

2′,7′-Dichlorofluorescein diacetate (DCFH-DA) is a widely used fluorogenic probe for detecting ROS and assessing overall oxidative stress in cells (Zhu et al. 2017; Murphy et al. 2022). This membrane-permeable compound diffuses into cells, where it is hydrolyzed by intracellular esterases to form the non-fluorescent 2′7′-dichlorofluorescin (DCFH), as depicted in Fig. 4A. Once formed, DCFH is retained within the cell and reacts with various ROS to generate the highly fluorescent 2′,7′-dichlorofluorescein (DCF), which can be quantified using flow cytometry, fluorescence plate readers, or fluorescence microscopy (Zhu et al. 2017; Ng and Ooi 2021; Yu et al. 2021). DCF fluorescence is typically measured at 538 nm following excitation at 488 nm.

DCFH-DA assays are commonly used as an initial, semi-quantitative screening probe to compare overall ROS generation under normoxic and hypoxic conditions (Lv et al. 2018; Teng et al. 2025). Under normoxia, increased fluorescence generally reflects the global oxidative burst induced by irradiation and is often used to validate light-triggered ROS production and evaluate the overall photosensitizing performance of a PS (Lv et al. 2018). In this context, DCFH-DA measurements are commonly complemented by more selective probes, such as dihydroethidium (DHE) for O_2_^•^⁻ (Yu et al. 2022; An et al. 2022) or Singlet Oxygen Sensor Green (SOSG) for ^1^O_2_ (Yu et al. 2022), in order to distinguish between Type I and Type II pathways. Normoxic experiments are also frequently used to establish baseline photochemical parameters, including total ROS generation, ^1^O_2_ quantum yield, and cellular phototoxicity. For example, Lv et al*.* (2018) showed that the cyclometalated Ru(II) complex Ru2 generated higher total ROS under normoxic conditions despite a relatively modest ^1^O_2_ yield, supporting the view that some PSs can act as efficient Type I biased ROS generators even when Type II activity is limited (Lv et al. 2018).

Under hypoxia, persistence of DCF fluorescence may indicate low-O_2_-dependent ROS generation pathways associated with Type I photochemistry (Li et al. 2022b; An et al. 2022). This comparison is particularly relevant because hypoxic tumor environments strongly limit classical Type II PDT, which depends on molecular oxygen for ^1^O_2_ production (Lv et al. 2018; Li et al. 2022b). Moreover, irradiation itself can aggravate local oxygen depletion through continuous O_2_ consumption (Lv et al. 2018). Consequently, comparing DCFH-DA signals under normoxia and hypoxia has become a useful strategy for evaluating whether a PS retains photodynamic activity under low oxygen conditions (Lv et al. 2018). At least one study reported measurable DCF fluorescence at 1% O_2_, suggesting that electron-transfer mediated ROS generation pathways remain operative under hypoxia (An et al. 2022).

It is important to note that DCFH-DA is a broad-spectrum indicator of oxidative stress and lacks specificity for individual reactive species. It does not selectively detect H_2_O_2_, lipid peroxides, ^1^O_2_, or O_2_^•^⁻. Therefore, the inclusion of appropriate controls is recommended. For instance, oxidative activity can be simulated using H_2_O_2_ or tert-butyl hydroperoxide (TBHP), depending on the cell type and analysis duration (Eruslanov and Kusmartsev 2010; Ng and Ooi 2021). Negative controls designed to suppress the ROS-generating process, including gene knockouts, knockdowns, inhibitors, or radical scavengers can help confirm which ROS are responsible for the oxidative damage (Murphy et al. 2022). Additionally, although DCFH-DA assays are widely employed in PDT studies, they are generally considered semi-quantitative rather than truly quantitative measurements of ROS production. Recent reviews on Type I PDT have further emphasized that robust quantitative methodologies for assessing Type I ROS generation and quantum yields are still lacking (Lv et al. 2018; Chen et al. 2021b). To complement its use, other probes have been applied in an attempt to detect specific reactive species (Eruslanov and Kusmartsev 2010).

### Detection of superoxide anion (O_2_^•⁻^)

O_2_^•^⁻ exhibits selective reactivity, as it does not readily oxidize most biological molecules, but can specifically damage certain enzymes containing Fe–S clusters (Murphy et al. 2022). In simplified systems, O_2_^•^⁻ quantified through the SOD-inhibitable reduction of cytochrome c (Nauseef 2014). However, in cellular systems, detection is commonly achieved using the fluorescence generated by the oxidation of dihydroethidium (DHE) (Fig. 4B) or its mitochondria-targeted analogue, MitoSOX (Murphy et al. 2022).

Hydroethidine (HE)-based probes are widely employed for intracellular O_2_^•^⁻ detection (Bilski et al. 2009; Price and Kessel 2010; Zielonka and Kalyanaraman 2010; Kessel et al. 2011; Kalyanaraman et al. 2014; Ortega-Villasante et al. 2018). However, their specificity is often limited, as these dyes can also react with other ROS, including ^1^O_2_, •OH, and H_2_O_2_ (Bilski et al. 2009; Zielonka and Kalyanaraman 2010; Ortega-Villasante et al. 2018). Consequently, the resulting oxidation products may not exclusively reflect O_2_^•^⁻ activity. Currently, high-performance liquid chromatography (HPLC) is considered the most reliable method for identifying specific HE oxidation products (Kalyanaraman et al. 2014), since fluorescence microscopy alone does not allow definitive discrimination of intracellular O_2_^•^⁻ generation (Zielonka and Kalyanaraman 2010).

In the context of PDT, detection of O_2_^•^⁻ has become particularly relevant for distinguishing hypoxia-tolerant Type I pathways from conventional oxygen-dependent Type II mechanisms (Li et al. 2022b; Teng et al. 2025). Under normoxia, DHE fluorescence is commonly employed to validate the ability of a PS to generate O_2_^•^⁻ through electron-transfer reactions (Li et al. 2020; Yu et al. 2022). In these conditions, O_2_^•^⁻ measurements are frequently interpreted together with total ROS and ^1^O_2_ assays to establish mechanistic reference points for PS activity (Li et al. 2020; Yu et al. 2022). Under hypoxia, O_2_^•^⁻ detection becomes especially informative because several Type I PS retain O_2_^•^⁻ generation even when ^1^O_2_ production is strongly suppressed by oxygen limitation (Yu et al. 2022; An et al. 2022). In some cases, DHE signals persist or even increase under low oxygen conditions, supporting the interpretation that electron-transfer-mediated ROS pathways remain active in hypoxic environments (Li et al. 2020; An et al. 2022).

Mamalis et al*.* (2016) investigated intracellular O_2_^•^⁻ generation in human dermal fibroblasts treated with aminolevulinic acid (ALA)-based PDT, employing DHE as a probe due to its relative selectivity for O_2_^•^⁻. Cells were pre-incubated with ALA, followed by DHE loading and subsequent irradiation. O_2_^•⁻^ production was then quantified by flow cytometry, using fluorescence intensity as a proxy for intracellular O_2_^•^⁻ levels (Mamalis et al. 2016). Similarly, Dolgachev et al*.* (2005) evaluated O_2_^•^⁻ formation in cells subjected to PDT with a phthalocyanine-based PS (Dolgachev et al. 2005). More recently, Type I PSs such as SORgenTAM (Li et al. 2020; Chen et al. 2021b) and NI-TA (Yu et al. 2022) demonstrated sustained O_2_^•^⁻ generation under hypoxic conditions, reinforcing the importance of O_2_^•⁻^ detection as a mechanistic indicator of a low O_2_-dependent mechanism.

The Organization for Economic Cooperation and Development (OECD) adopted Test Guideline No. 495: *Reactive Oxygen Species (ROS) Assay for Photoreactivity*, last reviewed and confirmed in 2019, which recommends the nitroblue tetrazolium (NBT) assay for detecting O_2_⁻^•^. In this assay, O_2_⁻^•^ reduces NBT via a one-electron transfer reaction, forming a partially reduced monoformazan derivative (NBT⁺), as illustrated in Fig. 4C (OECD 2024). This stable intermediate absorbs light at 560 nm, allowing spectrophotometric quantification of O_2_⁻^•^ (OECD 2024).

In the context of PDT, some NBT assays were conducted in solution rather than in cell-based systems, often using NADH as a positive control to induce O_2_⁻^•^ generation (Ikeda et al. 2017; Wen et al. 2017; Puttaswamy et al. 2023). In contrast, other studies applied the assay in cellular environments. For example, Malacarne (2023) and Panzarini et al. (2011) monitored ROS production in cells following PDT treatment using NBT (Panzarini et al. 2011; Malacarne et al. 2023). Kessel (2011) also employed NBT to detect intracellular O_2_⁻^•^ formation, reporting higher selectivity compared to DHE (Kessel et al. 2011). Despite its sensitivity to O_2_⁻^•^ (and to a lesser extent to ^•^OH), the non-fluorescent nature of NBT limits its utility in fluorescence-based applications (Kessel et al. 2011). Nevertheless, because O_2_⁻^•^ generation is frequently preserved under hypoxia in Type I PDT systems, NBT assays may provide valuable complementary information when comparing ROS production under normoxic and hypoxic conditions.

### Detection of hydroxyl radical (^•^OH)

^•^OH radicals are highly reactive radical species that interact indiscriminately with nearby molecules at nearly diffusion-controlled rates (Murphy et al. 2022). To detect these radicals, Setsukinai et al*.* (2003) developed two fluorescent probes: 2-[6-(4’-hydroxy)phenoxy-3H-xanthen-3-on-9-yl]benzoic acid (HPF) and 2-[6-(4’-amino)phenoxy-3H-xanthen-3-on-9-yl]benzoic acid (APF) (Setsukinai et al. 2003). Initially non-fluorescent, these probes emit fluorescence upon reaction with ^•^OH, forming stable O-dearylation products (Fig. 4D). The use of hydroxyl radical scavengers, such as dimethyl sulfoxide (DMSO), significantly reduces fluorescence, supporting their preferential reactivity with ^•^OH. Due to their stability and resistance to autooxidation, HPF and APF are especially suited for use in cellular environments (Setsukinai et al. 2003; Gomes et al. 2005). Although APF is considered relatively selective for ^•^OH, particularly in the presence of HRP, studies have also suggested that it may interact with ^1^O_2_ as well (Price and Kessel 2010).

The detection of ^•^OH in PDT-treated systems has become increasingly relevant in studies investigating Type I photochemical pathways under hypoxia (Chen et al. 2021b; Teng et al. 2025). Unlike ^1^O_2_ generation, which strongly depends on molecular oxygen, ^•^OH may arise from downstream radical cascades involving O_2_^•⁻^ disproportionation, Haber–Weiss reactions, and Fenton-type processes (Ochsner 1997; Yu et al. 2022; Li et al. 2022b). Consequently, the persistence of ^•^OH generation under low oxygen conditions is often interpreted as evidence of “oxygen-independent” or partially oxygen-independent ROS production (Lv et al. 2018; Li et al. 2022b).

The detection of ROS in PDT-treated systems has been explored using APF, a cell-permeable probe sensitive to highly reactive species such as ^•^OH and ^1^O_2_. Price et al*.* (2009) demonstrated its utility in mammalian cells by incubating them with both the PS and APF, with ^•^OH scavengers like DMSO as a negative control. Following irradiation, fluorescence microscopy confirmed ROS generation, and the inclusion of DMSO enabled the assessment of ^•^OH involvement specifically (Price et al. 2009). In another approach applied to microbial systems, Sułek et al*.* (2020) employed APF in bacterial PDT studies using dual-color flow cytometry. Bacteria were incubated with a porphyrin-based PS and APF, then irradiated, allowing ROS detection and characterization of oxidative stress responses via flow cytometry (Sułek et al. 2020).

Type I PDT systems active under hypoxia have demonstrated significant ^•^OH formation through downstream radical cascades. Lv et al*.* (2018) reported that the cyclometalated Ru(II) PS Ru2 retained PDT efficacy under both normoxia and hypoxia, and DMPO-EPR detected DMPO-OH adducts, supporting ^•^OH as a major contributor to phototoxicity (Lv et al. 2018). Similarly, NI-TA was proposed to generate O_2_⁻^•^ that feeds SOD, Haber–Weiss, and Fenton chemistry to produce ^•^OH under hypoxia (Yu et al. 2022). In that study, HPF was used as the ^•^OH fluorescent probe, while DMPO was used for EPR detection of O_2_⁻^•^ (Yu et al. 2022).

### Detection of singlet oxygen (^1^O_2_) in cells

Two singlet states of O_2_ are known, but only the ^1^Δg state, although not a free radical, is of major biological significance. This singlet configuration makes O_2_ far more reactive than its ground-state triplet form. ^1^O_2_ can be generated via photosensitization, where a PS absorbs light and transfers the energy to ground-state O_2_, as well as through chemical reactions involving HOO^•^ radicals, among others (Murphy et al. 2022).

Detecting ^1^O_2_ is challenging due to its high reactivity and the lack of selectivity of most probes. However, Singlet Oxygen Sensor Green (SOSG) has emerged as a relatively selective probe. In aqueous solution, SOSG emits weak blue fluorescence, initially quenched by photo-induced electron transfer from the anthracene donor to the fluorophore. Upon reaction with ^1^O_2_, a stable endoperoxide is formed (Fig. 4E), which suppresses this quenching and leads to a strong green fluorescence with emission peaks at 525–536 nm (excitation: 375 and 393 nm) (Ragàs et al. 2009; Kim et al. 2013b).

SOSG has been widely used to detect intracellular ^1^O_2_ in vitro using fluorescence microscopy (Flors et al. 2006; Hatz et al. 2007; Gollmer et al. 2011; Prasad et al. 2018; Chen et al. 2021a, 2025). Although traditionally considered cell-impermeable, evidence suggests that SOSG can be internalized under specific conditions, such as cell stress or in the absence of proteins (Peng et al. 2020). For example, SOSG has been incorporated into HGC-27 gastric cancer cells to detect ^1^O_2_ generated by polypyridyl Os(II) complexes, using a concentration of 2.5 µM in DMSO (Chen et al. 2025).

Similarly, SOSG uptake has been demonstrated in HeLa cells when cultured in standard maintenance medium (SMM), a protein-free culture medium (Hatz et al. 2007). In contrast, no significant uptake was observed when cells were maintained in conventional Dulbecco’s Modified Eagle’s Medium (DMEM) supplemented with fetal calf serum and antibiotics. This suggests that SOSG binds strongly to proteins, which can inhibit its internalization and alter its intracellular behavior (Prasad et al. 2018). Therefore, SOSG has been utilized both in cell-free systems, where potassium iodide served as a negative control (Wen et al. 2017), and in cellular environments, where its application was combined with confocal fluorescence microscopy to monitor intracellular ^1^O_2_ generation (Chen et al. 2015).

In PDT studies, ^1^O_2_ detection under normoxic conditions is commonly used to evaluate classical Type II photochemical activity and determine baseline photodynamic efficiency (Li et al. 2022b). Under air-saturated conditions, strong SOSG signals are generally interpreted as evidence of efficient energy transfer from the excited PS to molecular oxygen (Yu et al. 2022). Consequently, normoxic experiments are frequently used to determine ^1^O_2_ quantum yields and compare the Type II performance of different PSs (Lv et al. 2018).

In contrast, hypoxic experiments serve as an important stress test for oxygen dependence in PDT systems (Lv et al. 2018; An et al. 2022). Because Type II PDT relies directly on molecular oxygen, ^1^O_2_ generation is typically attenuated under low oxygen conditions, and irradiation itself may further aggravate oxygen depletion (Lv et al. 2018; Li et al. 2020). In some hypoxia-tolerant PDT systems, reduced SOSG fluorescence under hypoxia was accompanied by persistent DHE or DCFH-DA signals, supporting the interpretation that ROS generation shifted from Type II to Type I pathways (Yu et al. 2022; An et al. 2022). These observations reinforce the idea that ^1^O_2_ production alone is insufficient to evaluate PDT efficiency under hypoxic tumor conditions (Lv et al. 2018; Yu et al. 2022; Li et al. 2022b).

The Organization for Economic Cooperation and Development (OECD) adopted Test Guideline No. 495: *ROS Assay for Photoreactivity* last reviewed and confirmed in 2019 (OECD 2024), which recommends the p-nitrosodimethylamine (RNO) assay for detection of ^1^O_2_. This method is based on the procedure developed by Kraljic and El Mohsni, in which the generation of ^1^O_2_ is monitored by the spectrophotometric measurement of RNO bleaching, observed as a decrease in absorbance at 440 nm (Kraljić and Mohsni 1978; Onoue et al. 2008).

Although ^1^O_2_ does not directly react with RNO, the bleaching effect results from the interaction of ^1^O_2_ with an imidazole ring, leading to the formation of a trans-annular peroxide intermediate. This reactive intermediate subsequently oxidizes RNO, causing its decolorization (Fig. 4F). In the context of PDT, this assay is typically performed in aqueous solution in the presence of histidine, which serves as the imidazole source (Liu et al. 2008; Kim et al. 2013a; Simaremare et al. 2015; Urazaliyeva et al. 2024).

## Cellular uptake and subcellular localization

Cellular uptake and subcellular localization of PSs are key determinants of PDT efficacy, as they govern both intracellular PS concentration and proximity to critical biomolecular targets (Castano et al. 2005; Benov 2015). Given the short diffusion radius and lifetime of ROS, oxidative damage is largely confined to the immediate microenvironment of the photoexcited PS, making this spatial PS location particularly important for treatment outcome (Tsubone et al. 2019a, b, 2021).

Many PSs inherently fluoresce upon exposure to specific excitation wavelengths, rendering flow cytometry (Salvati et al. 2018) or microplate readers (Shin et al. 2022), both effective methods for quantifying their uptake by mammalian cells or bacteria ​(Kiesslich et al. 2013). When a PS lacks intrinsic fluorescence, it can be conjugated to a fluorescent marker to facilitate detection and analysis. This process involves measuring the fluorescence intensity of individual cells after incubation with the PS for a defined period. Critical experimental variables, such as PS concentration, incubation duration, and environmental conditions (e.g., hypoxia, temperature, pH), must be optimized based on the specific goals and properties of the PS.

Fluorescence intensity is directly proportional to the number of PS accumulated within each cell. The flow cytometry data are typically represented in histograms or scatter plots, where the *x*-axis indicates fluorescence intensity (reflecting PS uptake), and the *y*-axis represents cell count or frequency. Cells with higher fluorescence intensities indicate greater uptake of the PS. The data can be used to quantify the percentage of cells that have internalized the PS and to compare uptake levels across different cell types or experimental conditions, such as normoxia or hypoxia (Kiesslich et al. 2013).

Confocal laser scanning fluorescence microscopy has improved the analysis of PS subcellular localization, offering higher sensitivity and spatial resolution compared to nonconfocal methods (Woodburn et al. 1991). Colocalization with organelle-specific fluorescent probes—whose emission spectra differ from that of the PS—allows precise identification of the accumulation site (Tsubone et al. 2019b). These probes can also highlight damaged regions postirradiation, supporting detailed assessment of PDT effects. Excitation and emission wavelengths must be carefully selected based on the optical properties of both the PS and the probes (Tsubone et al. 2020).

PSs, in general, can localize in mitochondria, lysosomes, endoplasmic reticulum, Golgi apparatus and plasma membranes (Kurutos et al. 2022). The probes more commonly used in studies are: mitochondria (Mito-tracker™ Red, MitoTracker™ Deep Red FM, MitoTracker™ Orange or rhodamine 123) (Teiten et al. 2003; Li et al. 2018c; Martins et al. 2019), lysosomes (LysoTracker™ Green DND-26 or LysoTracker™ Red DND-99) (Kiesslich et al. 2013), endoplasmic reticulum (ER Tracker Blue/White™ or DiOC_6_) (Carneiro et al. 2018), and golgi apparatus (BODIPY® FL C_5_-ceramide, BPC) (Teiten et al. 2003).

For the analysis, selected cell lines should be cultured in an appropriate growth medium and incubated under controlled conditions (normoxia or hypoxia) until they reach the desired confluence. After achieving the proper confluence, the cells must be treated with the PS at the established concentration and incubated for the necessary period to allow for adequate internalization. Following this, specific fluorescent probes targeting organelles of interest should be incubated with the cells. Once the incubation is complete, confocal microscopy should be employed to analyze the PS and the organelle markers. This enables overlay assessment, providing insights into the subcellular localization of the PS. For optimal results in confocal microscopy, it is essential to select the appropriate laser wavelength for excitation based on the fluorescent probes used and an emission filter set compatible with these probes. The resulting images will reveal the spatial distribution of the PSs within the cell, facilitating the analysis of their subcellular localization (Woodburn et al. 1991; Moserova and Kralova 2012; Chota et al. 2024).

To interpret these data, the PS images should be overlaid (or colocalized) with images of the organelle-specific probes. Visual colocalization is initially identified by the appearance of structures whose color reflects the combined contribution of both species when the images of each probe are overlaid (or “merged”). For example, if the PS exhibits red fluorescence and the organelle-specific probe shows green fluorescence, colocalization will be observed as an orange or yellow color resulting from the combination of both fluorescence signals (Teiten et al. 2003). The obtained images can then be analyzed using ImageJ or ZenSoftware to quantify the colocalization between the PS and organelle-specific probes ​(Schneider et al. 2012; Pucelik et al. 2020). Although visual inspection of merged fluorescence images provides an initial indication of PS localization, qualitative assessment alone may be subjective. Therefore, colocalization should be supported by quantitative metrics that objectively describe the spatial overlap between the PS and organelle-specific markers.

For quantitative analysis of colocalization, ImageJ plugins such as Colocalization Test (Fay et al. 1997) and Colocalization Threshold ​(Costes et al. 2004) can be employed. The Pearson correlation coefficient (PCC) measures the correlation between the fluorescence intensities of the PS and the organelle marker on a pixel-by-pixel basis, providing an estimate of the overall degree of colocalization. In contrast, Manders’ colocalization coefficients (M1 and M2) quantify the fraction of one fluorophore overlapping with the other, thereby indicating the proportion of PS associated with a given organelle and vice versa. Because PCC and Manders’ coefficients provide complementary information, they are commonly reported together to objectively support the qualitative interpretation of merged confocal images (Dunn et al. 2011).

Most protocols to measure cellular uptake and subcellular localization of PS are often characterized under normoxic culture conditions, implicitly assuming that both processes remain unchanged at reduced oxygen levels. Although hypoxia does not directly modify the intrinsic physicochemical properties of PSs—such as charge, lipophilicity, or aggregation propensity—which remain the primary determinants of cellular entry and organelle targeting (MacDonald et al. 1999; Ezzeddine et al. 2013; Mallidi et al. 2016; Martinez De Pinillos Bayona et al. 2017; Chen et al. 2023), it influences overall PS accumulation and retention indirectly through hypoxia-induced cellular responses. Stabilization of hypoxia-inducible factor 1α (HIF-1α) drives extensive metabolic and transcriptional reprogramming (Infantino et al. 2021), including shifts lipid composition of membranes (Botto et al. 2008; Seo et al. 2022), modulates vesicular trafficking (Muñiz-García et al. 2022), and changes the expression of transporters and stress-response proteins (Parks et al. 2016). These changes can modulate passive diffusion and endocytic uptake pathways, thereby affecting the intracellular concentration and aggregation state of PSs without altering their chemical identity.

Hypoxia also affects subcellular localization primarily as a consequence of global cellular stress responses rather than through direct relocalization of the PS itself. Mitochondria, which are frequent targets for cationic and lipophilic PSs, undergo functional remodeling under hypoxia, including depolarization of the mitochondrial membrane potential (Δ*Ψ*_m_), structural fragmentation, and reduced oxidative activity (Fuhrmann and Brüne 2017). These changes can diminish mitochondrial accumulation of PSs that rely on Δ*Ψ*_m_-driven uptake, leading to attenuated Type II photochemistry and reduced phototoxicity (Lv et al. 2016b; An et al. 2022). In parallel, hypoxia-induced activation of autophagy alters vesicular trafficking and organelle turnover, potentially sequestering PSs into lysosomal (Lv et al. 2016b; An et al. 2022) or endoplasmic reticulum (Zhuang et al. 2020; Li et al. 2022c) compartments and modifying their effective photochemical microenvironment.

Methodologically, the techniques used to quantify uptake and localization in normoxia—such as fluorescence spectroscopy of cell lysates, flow cytometry, confocal microscopy, and colocalization with organelle-selective dyes—remain applicable under hypoxia, but their readouts must be interpreted with additional care. Fluorescence quenching by hypoxia-induced changes in protein or lipid environment, altered autofluorescence, and differences in probe behavior at low O_2_ can all bias apparent PS concentrations or colocalization coefficients (Peskova et al. 2021), particularly in 3D spheroids where light scattering and absorption are significant (Kucinska et al. 2021; Abolhassani et al. 2023). Whenever possible, uptake studies in hypoxic models should therefore combine quantitative approaches (for example, flow cytometry or lysate fluorescence calibrated to standard curves) with high-resolution imaging in 2D and 3D and should validate organelle assignment using multiple markers or genetically encoded reporters rather than a single commercial dye.

Finally, hypoxia-adapted or PDT-experienced cell populations may acquire stable changes in transporter expression and membrane organization that further reshape PS uptake profiles over time. In such systems, comparing PS accumulation and localization between parental and hypoxia-adapted lines, or between 2D monolayers and size-matched spheroids, can help distinguish the direct oxygen effects from longer-term adaptive remodeling. Integrating these uptake and localization measurements with oxygen-controlled ROS profiling and organelle-specific damage readouts is therefore crucial for mechanistically sound evaluation of PSs in hypoxia-relevant in vitro models.

## Oxidative damage to biomolecules under normoxia and hypoxia

Many methods are available to evaluate oxidative stress in biomolecules—proteins, lipids, and DNA—by detecting both primary (e.g., hydroperoxides, radicals) and secondary (e.g., aldehydes, carbonyls) oxidation products (Juan et al. 2021). During PDT, the ROS generated are primarily responsible for inducing oxidative damage to these biomolecules (Tsubone et al. 2019a), ultimately leading to cell death (Tsubone et al. 2021). However, hypoxia fundamentally alters which ROS predominate and, consequently, which biomolecular targets are most affected depending on subcellular localization.

Under normoxic conditions, Type II photochemistry dominates for most clinically used PSs, generating ^1^O_2_ that exhibits relatively selective reactivity toward electron-rich amino acid residues—particularly histidine, methionine, tryptophan, tyrosine, and cysteine—with rate constants exceeding 10^7^ dm^3^ mol^−1^ s^−1^ (Davies 2004). In contrast, under hypoxia, ^1^O_2_ formation is markedly suppressed due to limited ground-state oxygen availability, and Type I pathways involving direct electron or hydrogen transfer from the excited PS to substrates become comparatively more important (Davies 2004; Teng et al. 2025). The resulting radical species—O_2_^−•^, ^•^OH, and HOO^•^—are less selective and react with a broader range of biomolecular targets, initiating chain reactions that can propagate oxidative damage beyond the immediate vicinity of the photoexcited PS (Juan et al. 2021).

This mechanistic shift has direct implications for the pattern and extent of oxidative injury observed in hypoxic PDT models. Whereas ^1^O_2_-mediated damage tends to produce well-defined oxidation products on specific amino acid residues (Davies 2004; Ronsein et al. 2006; Riske et al. 2009), radical-driven oxidation under hypoxia can lead to widespread protein carbonylation, thiol oxidation across multiple sites, and—critically—enhanced lipid peroxidation via chain-propagating HOO^•^ radicals (Bacellar and Baptista 2019; Rezende et al. 2022). Recent studies on Type I PSs and PDT-induced ferroptosis-like cell death demonstrate that lipid hydroperoxide accumulation, glutathione depletion, and GPX4 inactivation can become dominant features of hypoxic PDT, even when conventional markers of ^1^O_2_-mediated damage are attenuated (Shui et al. 2021a; Qi et al. 2023).

### Photoinduced oxidative damage to proteins by PSs

Among the biomolecules mentioned, proteins are considered one of the main photodynamic targets, both due to their abundance—making up about 70% of the dry weight of cells—and due to their high reaction rates with ^1^O_2_ and other ROS (Szokalska et al. 2009). Under normoxia, the amino acid residues of cysteine, histidine, methionine, tyrosine, and tryptophan are the primary ones involved in protein photooxidation (Benov 2015). Since tryptophan and tyrosine are aromatic amino acid residues that emit intrinsic fluorescence around 350 nm in proteins (Lakowicz 1994), photosensitized protein oxidation can be evaluated by monitoring the decrease in fluorescence intensity (e.g., in HSA) (Hirakawa et al. 2020, 2022). As example, Hirakawa and collaborators (2022) evaluate the protein photodamage to HSA induced by two different water-soluble cationic phthalocyanine PSs—2,3,6,7,10,11,14,15-octaquiso-[N-methyl-(4-methylpyridinium-3-yl-oxy)phthalocyaninato] chloride gallium(III) iodide (GaPyPc) and 2,3,6,7,10,11,14,15-octaquiso-[N-methyl-(4-methylpyridinium-3-yl-oxy)phthalocyaninato] chloride zinc(II) iodide (ZnPyPc) in the presence of sodium azide (NaN_3_), which is used to quench the ^1^O_2_ generated during PDT application (Hirakawa et al. 2022).

Under hypoxia, however, the relative contribution of radical-mediated oxidation increases. Hydroxyl radicals (^•^OH) oxidize tryptophan to hydroxytryptophan, which is subsequently cleaved by residual oxygen into N-formylkynurenine—a pathway that can proceed even at low O_2_ levels and does not require ^1^O_2_. Similarly, cysteine thiol groups, which are highly susceptible to both ^1^O_2_ and radical attack, may undergo oxidation via different mechanisms depending on oxygen availability: ^1^O_2_ favors sulfenic acid formation, whereas ^•^OH and O_2_^−•^ can generate thiyl radicals that propagate disulfide cross-linking and mixed oxidation products (Davies 2004; Shui et al. 2021a).

**Fig. 5:**
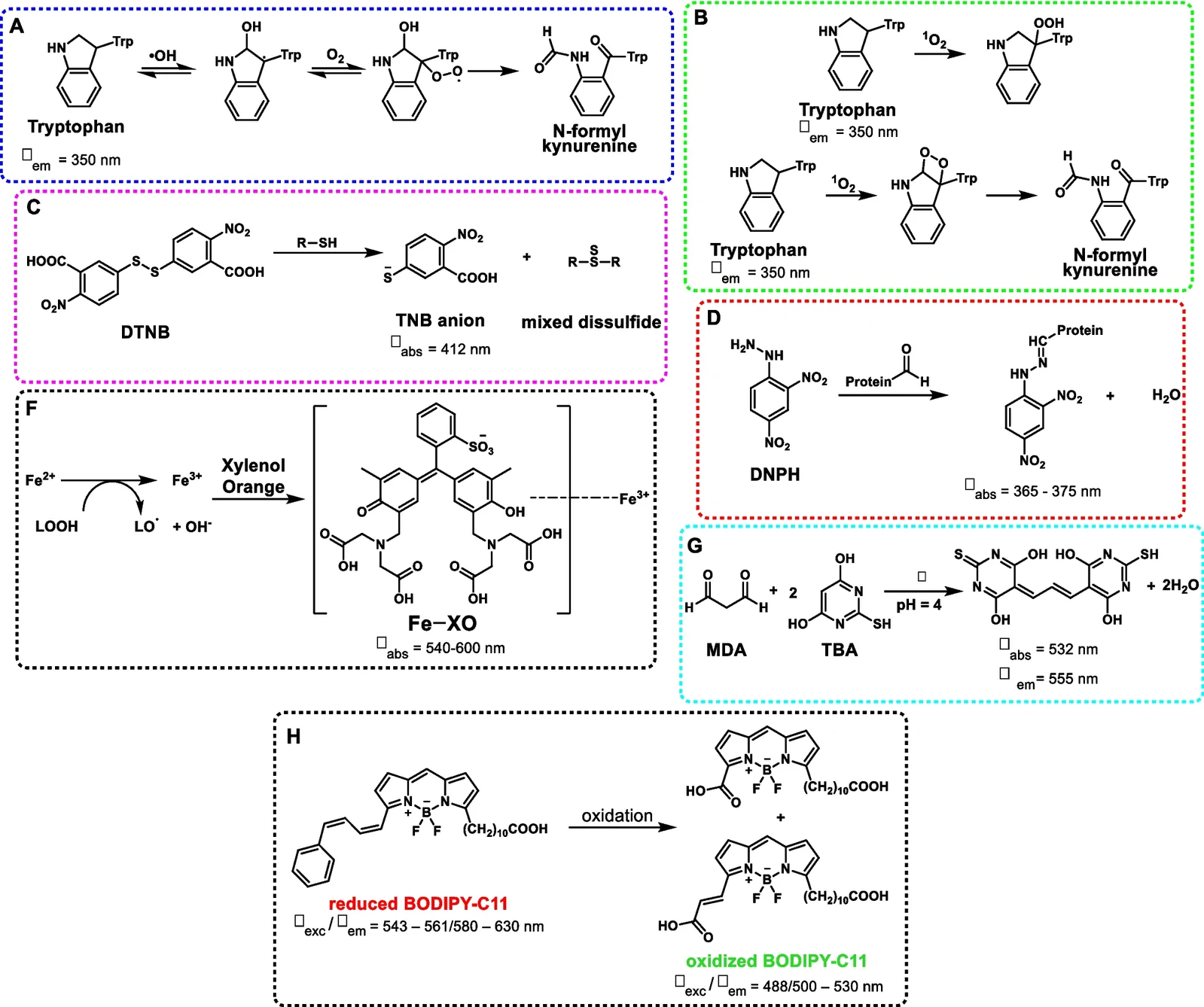
Schematic representation of the chemical reactions involved in the assessment of oxidative damage to biomolecules. Protein oxidation: (**A**) oxidation of tryptophan by hydroxyl radicals (•OH) and singlet oxygen (^1^O_2_), illustrating distinct oxidative pathways; (**B**) reactions underlying the DTNB assay for quantification of protein thiol oxidation through detection of free sulfhydryl groups; and (**C**) reactions involved in the DNPH assay for detection of protein carbonyl groups as markers of oxidative protein damage. Lipid peroxidation: (**D**) reactions underlying the FOX assay for detection of lipid hydroperoxides; (**E**) reactions involved in the TBARS assay for evaluation of lipid peroxidation through malondialdehyde (MDA) detection; and (**F**) oxidation reactions of BODIPY-C11 used to assess lipid peroxidation in vitro

Therefore, the photodamage to protein is induced by both ^1^O_2_ and electron transfer, and the oxidation of protein containing tryptophan via electron transfer can generate N-formylkynurenine (Fig. 5A). More specifically, hydroxyl radicals oxidize tryptophan to hydroxytryptophan, which is subsequently cleaved by oxygen into N-formylkynurenine, as shown in Fig. 5B ​ (Lowry et al. 1951).

Another approach to evaluate protein oxidative damage induced by photosensitization involves the detection or quantification of amino acid residues containing thiol groups, such as cysteine and methionine. A decrease in –SH group concentration following PDT treatment reflects the extent of protein oxidation (Saczko et al. 2004; Ion et al. 2012). The most widely used method for thiol quantification is the 5,5′-dithiobis(2-nitrobenzoic acid) assay, commonly known as Ellman’s reagent (referred to herein as DTNB assay) (Moan and Vistnes 1986; Rice-Evans et al. 1991; Daicoviciu et al. 2011), which will be discussed in detail in the next subsection (Sect. “Protein thiol groups or DTNB assay”). Protein photooxidation can also be assessed by measuring carbonyl contents. The 2,4-dinitrophenylhydrazine (DNPH) assay is a widely used method for measuring protein oxidation by detecting carbonyl groups in proteins (Tseng et al. 2009) and will also be described below (Sect. “DNPH assay”).

#### Protein thiol groups or DTNB assay

Thiol groups play a crucial role in the function of metabolic enzymes and cellular signaling processes that mediate responses to environmental factors. Among these, cysteine thiol groups are particularly important due to their reversible redox state, which directly influences structural, catalytic, and regulatory protein functions. However, these groups are highly susceptible to oxidation, which can lead to alterations in protein activity and cellular homeostasis (Moan and Vistnes 1986; Rice-Evans et al. 1991; Daicoviciu et al. 2011).

One of the most commonly used spectrophotometric methods for detecting thiol groups in both protein and non-protein molecules is the Ellman’s reagent assay (Rice-Evans et al. 1991). This assay utilizes 5,5′-dithiobis(2-nitrobenzoic acid) (DTNB), which undergoes a disulfide exchange reaction with thiol groups, producing the yellow-colored 5-thio-2-nitrobenzoate anion (TNB) (Fig. 5C). TNB exhibits a high molar absorption coefficient (*ε* = 13,600 M^−1^ cm^−1^ at 412 nm), enabling its concentration to be determined from absorbance measurements according to the Beer-Lambert law. Because one mole of TNB is produced for each reacting thiol group, the absorbance directly reflects the thiol concentration in the sample ​ (Rice-Evans et al. 1991). The reaction is highly specific for thiol groups and is most effective at pH 8.0–9.0, as lower pH values reduce the chromophore’s absorbance (Rice-Evans et al. 1991). Additionally, the rate of TNB production can be used to assess the accessibility of thiol groups within proteins, where lower reaction rates indicate sterically hindered or inaccessible thiol groups ​(Rice-Evans et al. 1991).

In PDT studies, protein thiol oxidation has been used as a marker of oxidative damage ​ (Moan and Vistnes 1986; Du et al. 2003; Daicoviciu et al. 2011). Ellman’s method is applied to evaluate PDT-induced modifications in protein thiol content in cells (Moan and Vistnes 1986; Daicoviciu et al. 2011), often in combination with Western Blot analysis. In these assays, cells are treated with a PS and light, followed by incubation with DTNB to detect changes in free thiol availability. A modified protocol by Schothorst (1982) involves exposing cells to DTNB for 20 min at 20 °C, a reduction from the standard 60-min incubation time. This adjustment allows for the detection of early thiol oxidation events before secondary reactions and cellular repair mechanisms can significantly alter results. Control experiments with non-irradiated samples showed comparable results for 20-min and 60-min incubations, supporting the validity of the shorter incubation time (Moan and Vistnes 1986; Daicoviciu et al. 2011).

In addition to whole-cell assays, thiol oxidation can be assessed in lysed cells (Moan and Vistnes 1986). Sonicated cell samples undergo centrifugation to remove debris before the absorbance of the supernatant is measured at 412 nm. Scanning the full absorption spectrum enables correction for potential background interference or scattering from detached cells. Moreover, assays performed at 4 °C yielded similar relative thiol oxidation values but were less effective due to incomplete color development, reinforcing the preference for a 20 °C incubation temperature in most experiments (Moan and Vistnes 1986).

The Ellman’s reagent (DTNB) assay remains applicable for detecting protein thiol oxidation under hypoxia, but interpretation requires caution (Salyha and Oliynyk 2023b). Because radical-mediated thiol oxidation may produce a heterogeneous mixture of products—including sulfenic, sulfinic, and sulfonic acids, as well as inter- and intramolecular disulfides—the net decrease in free –SH groups detected by DTNB may not fully reflect the complexity of oxidative modifications. Additionally, hypoxia-adapted cells often exhibit elevated glutathione (GSH) pools and enhanced thioredoxin/glutaredoxin activity, which can partially buffer or reverse early thiol oxidation events before assay readout (Zhang et al. 2022; Wolny et al. 2024). Whenever possible, DTNB-based thiol quantification should be complemented by mass spectrometry or specific antibody-based detection of oxidized cysteine products to capture the full spectrum of modifications induced by hypoxic PDT.

#### DNPH assay

Protein carbonylation is an early marker of oxidative stress, once it occurs before major structural changes (Tseng et al. 2009). Unlike other oxidative modifications, such as thiol oxidation, protein carbonylation is irreversible, making it a reliable indicator of oxidative stress ​(Tseng et al. 2009). Therefore, protein carbonylation measured by the DNPH assay provides a robust and irreversible marker of oxidative protein damage that is equally relevant under normoxia and hypoxia. Importantly, carbonyl formation can arise from both ^1^O_2_-mediated and radical-mediated pathways: direct oxidation of lysine, arginine, proline, and threonine side chains (Juan et al. 2021), as well as secondary modifications by reactive aldehydes (e.g., malondialdehyde, 4-hydroxynonenal) generated during lipid peroxidation (Rezende et al. 2022).

As initially reported by Levine (Levine et al. 1994), the determination of carbonyl groups is performed using 2,4-dinitrophenylhydrazine (DNPH), which reacts with carbonyl groups, forming 2,4-dinitrophenylhydrazone (DNP)—Fig. 5D. Carbonyl content is measured around 360 nm, using an extinction coefficient of 22,000 M^−1^ cm^−1^ (Levine et al. 1994), allowing quantifying protein oxidation.

This method is applicable to both isolated proteins and cellular proteins. In cellular analysis, proteins are extracted by lysing cells through mild sonication, followed by centrifugation to remove insoluble debris. The resulting supernatant is divided into two aliquots: one reacts with DNPH to detect carbonyl groups, while the other serves as a control. Samples are then precipitated with trichloroacetic acid (TCA), washed with ethanol/ethyl acetate, and dissolved in guanidinium hydrochloride for spectrophotometric analysis.

In PDT studies, protein carbonyl analysis has been applied to bacteria (Tseng et al. 2009). Following photodynamic inactivation (PDI), bacterial cells are disrupted by sonication to obtain crude extracts for analysis. The assay follows the DNPH-labeling procedure, where crude extracts are incubated DNPH in HCl for 1 h at 37 °C. Protein blanks are prepared using HCl alone. After incubation, proteins are precipitated with 10% TCA, washed three times with ethanol/ethyl acetate (1:1) to remove excess DNPH, and dissolved in guanidine hydrochloride at 37 °C for 30 min. Carbonyl content is then quantified using absorbance technique (Tseng et al. 2009).

Similarly, in plasma protein carbonyl analysis, DNPH-based detection can also be used after PDT (Du et al. 2003). Plasma samples are incubated with DNPH in HCl for 1 h at room temperature in the dark. After protein precipitation with TCA and centrifugation, the washed protein pellet is dissolved in 6 M guanidine hydrochloride, and absorbance is measured at 360 nm. Protein concentration in each sample is estimated by absorbance readings at 280 nm, using a bovine serum albumin standard curve. Protein carbonyls values are then expressed as nmol carbonyl per mg of protein (Du et al. 2003).

In conclusion, protein carbonylation measurement is a tool for assessing oxidative damage across different biological contexts, including cellular proteins, plasma, and bacterial systems in PDT studies. Its irreversible nature makes it a robust biomarker for oxidative stress, facilitating research on oxidative damage and therapeutic interventions.

### Photoinduced oxidative damage to lipids by PSs

**Fig. 6:**
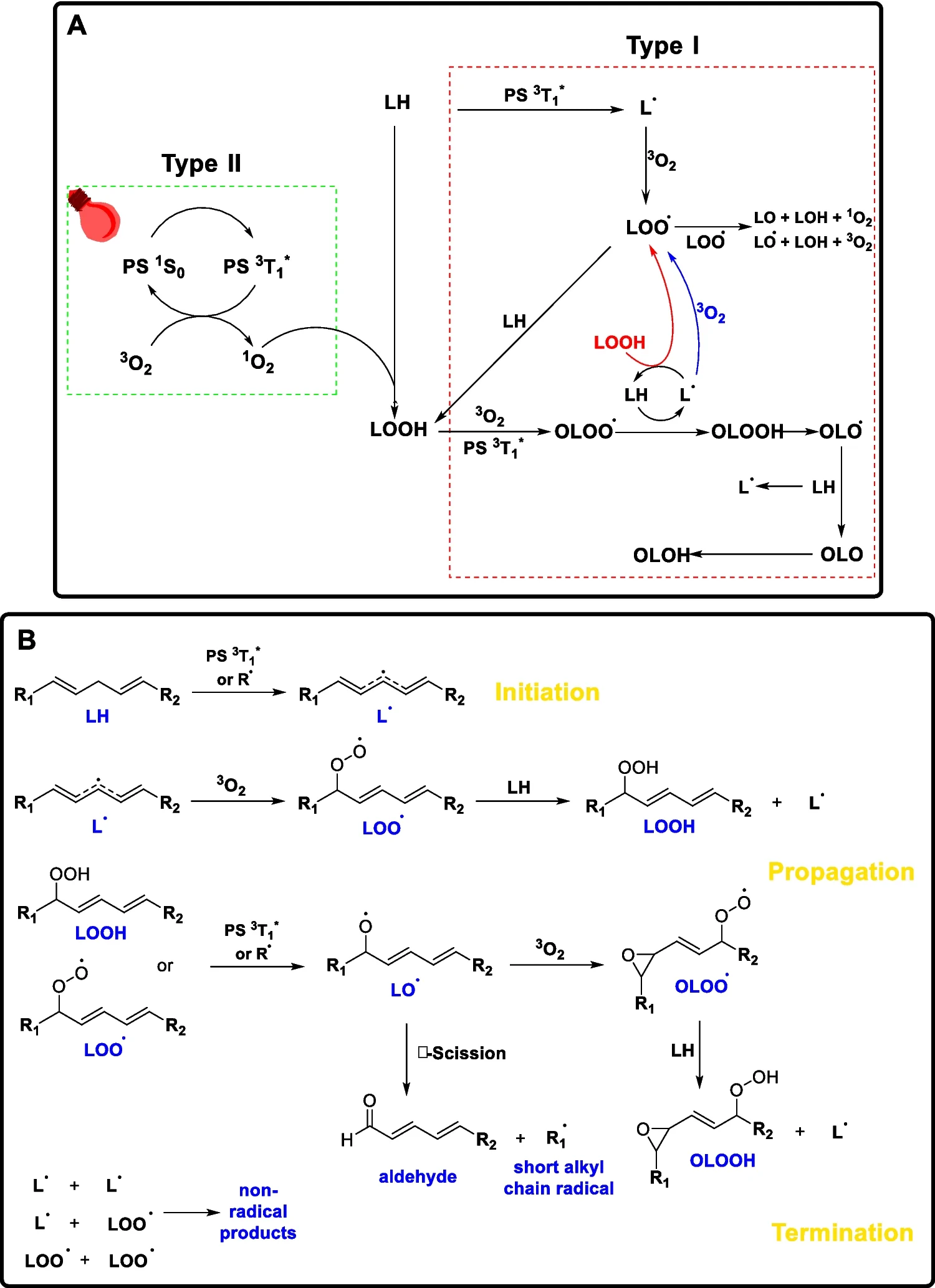
Schematic representation of (**A**) Type I and Type II photoinduced lipid oxidation pathways occurring in biological membranes. Abbreviations: LH, unsaturated lipid; LOOH, lipid hydroperoxide; OLOOH, epoxyallylic hydroperoxide; OLOH, epoxyallylic alcohol; LO, lipid ketone or aldehyde; LOH, lipid alcohol; L^•^, lipid radical; LOO^•^, peroxyl radical; OLOO^•^, epoxyallylic peroxyl radical; OLO^•^, epoxyallylic oxyl radical; and LO^•^, alkoxyl radical. PSs in the ground, excited triplet, and excited singlet states are represented by PS ^1^S_0_, PS ^3^T_1_^*^, and PS ^1^S_1_^*^, respectively. **B** General stages involved in the lipid peroxidation radical chain reaction, including initiation, propagation, and termination processes

Lipid peroxidation is one of the most frequently used indirect indicators of free radical activity in cells, involving the degradation of polyunsaturated fatty acids (PUFAs) in membrane phospholipids, lipoproteins, and other lipid structures (Daicoviciu et al. 2011). Among various cellular targets, lipid peroxidation is particularly harmful because its byproducts promote the rapid propagation of free radicals, amplifying oxidative damage (Saczko et al. 2004). This complex process generates a variety of stable yet toxic oxidized biomolecules, which can severely compromise membrane integrity (Daicoviciu et al. 2011). Under normoxia and Type II PS, ^1^O_2_ reacts directly with PUFA double bonds via a concerted “ene” reaction to form lipid hydroperoxides (LOOHs) without generating free radical intermediates—a non-chain-propagating mechanism that produces relatively defined hydroperoxide isomers (Fig. 6A) (Bacellar and Baptista 2019; Rezende et al. 2022).

Under hypoxia, Type I photochemistry becomes comparatively more important, and radical-initiated lipid peroxidation can dominate. In this pathway, abstraction of a bis-allylic hydrogen by ^•^OH or HOO^•^ radicals generates a carbon-centered lipid radical (L^•^), which reacts with residual O_2_ to form a peroxyl radical (LOO^•^) that propagates the chain by abstracting hydrogen from adjacent PUFAs (Fig. 6B) (Bacellar and Baptista 2019; Rezende et al. 2022). This chain reaction can amplify oxidative damage beyond the immediate site of PS localization and generates a broader spectrum of primary (hydroperoxides) and secondary (aldehydes, epoxides) products compared with ^1^O_2_-mediated peroxidation (Fig. 6). Recent work linking PDT to ferroptosis—an iron-dependent form of cell death driven by uncontrolled lipid peroxidation—highlights the biological relevance of this pathway in hypoxic settings. Type I PSs that generate O_2_^−•^ and ^•^OH have been shown to deplete GSH, inactivate GPX4, and trigger ferroptosis-like death in hypoxic tumor cells, providing a mechanistic rationale for sustained PDT efficacy under low oxygen conditions (Shui et al. 2021a; Qi et al. 2023). Notably, even at low O_2_ concentrations (< 2%), sufficient molecular oxygen may remain to sustain LOO^•^ radical formation and chain propagation, meaning that Type I–initiated lipid peroxidation can proceed under conditions where ^1^O_2_ generation is severely compromised (Shui et al. 2021a). Some reactive intermediates and end-products formed during photosensitized lipid oxidation exhibit inherent absorbance and/or fluorescence, allowing their detection and quantification through colorimetric or fluorometric methods, as outlined below.

Widely used assays such as FOX (*F*errous *O*xidation−*X*ylenol orange), thiobarbituric acid reactive substances (TBARS) or iodometric methods are primarily sensitive to secondary or stable lipid peroxidation products and may underestimate or misrepresent lipid damage generated via Type I mechanisms (Rubio and Cerón 2021; Murphy et al. 2022). Fluorescent probes such as BODIPY-C11 provide improved spatial resolution but remain sensitive to probe localization (Greene et al. 2018). Accordingly, interpretation of lipid oxidation data in hypoxic PDT models requires careful consideration of both oxygen tension and photochemical pathway. Combining multiple readouts—such as live-cell imaging, membrane integrity assays, and time-resolved measurements—can help distinguish between reduced oxidation efficiency and altered oxidative mechanisms.

#### FOX assay

The FOX (*F*errous *O*xidation−*X*ylenol orange) assay relies on the oxidation of ferrous ions (Fe^2+^) to ferric ions (Fe^3^⁺) by hydroperoxides under acidic conditions at room temperature (Fig. 5F) (Niki 2009, 2014). The dye Xylenol Orange binds to Fe^3^⁺, forming a chromophore complex with strong absorbance in the 540–600 nm range, which can be detected spectrophotometrically. Since hydroperoxide measurement is linked to free radicals and other reactive oxygen species, it serves as a key indicator of oxidative stress in biological samples (Halliwell and Chirico 1993; Bou et al. 2008). Therefore, its quantification provides an early and precise assessment of oxidative status (Bou et al. 2008). Additionally, this method is applicable to the determination of lipid hydroperoxides in liposomes, plasma, and lipoproteins (Jiang et al. 1991, 1992; Nourooz-Zadeh et al. 1994; Wolff 1994).

The FOX assay has two main versions. The first, FOX-1, uses an aqueous reagent containing sorbitol, which enhances the detection of water-soluble hydroperoxides such as hydrogen peroxide, butyl, and cumyl hydroperoxide (Jiang et al. 1991; Nourooz-Zadeh et al. 1994; Bleau et al. 1998; Banerjee et al. 2004; Cheeseman 2006). However, it is unsuitable for measuring low levels of lipid-soluble hydroperoxides in the presence of non-peroxidized lipids. To address this, FOX-2 was developed, utilizing a methanolic solution (90%, v/v) with butylated hydroxytoluene (BHT) as an antioxidant to prevent artificial oxidation. This version, widely applied to liposomes, cells and tissues, offers greater versatility ​(Hermes-Lima et al. 1995; Arab and Steghens 2004; Ayuyan and Cohen 2006; Fukuzawa et al. 2006; Bou et al. 2008; Angeli et al. 2011).

When quantifying hydroperoxides using the FOX method in PDT studies, special attention should be given to pigments that strongly absorb within the same spectral range as the Fe–XO complex. For example, anthocyanins at low pH (490–550 nm) and chlorophyll derivatives (with absorbance peaks at 400–455 and 645–665 nm) exhibit spectra spanning the orange to blue regions (Wrolstad et al. 2005; Bou et al. 2008). While these compounds do not strongly overlap with the Fe–XO absorbance range, high concentrations can still interfere with the assay, potentially affecting result accuracy.

The FOX-2 assay has been applied in PDT studies using PSs that induce photooxidation via a Type I mechanism (Vignoni et al. 2018; Sosa et al. 2023). This process involves the direct interaction of an excited-state PS, typically in its triplet state, with a target molecule or substrate. Through electron transfer or hydrogen abstraction, radicals are generated, which then participate in various competitive reactions, ultimately forming stable oxidized photoproducts (Sosa et al. 2023).

This mechanism follows a contact-dependent pathway, requiring direct interaction between the excited PS and the substrate, leading to significant membrane damage (Vignoni et al. 2018; Sosa et al. 2023). Increasing the lipophilicity of these PSs enhances their affinity for biomembranes, thereby promoting lipid hydroperoxide formation and further compromising membrane integrity ​(Sosa et al. 2023).

Other studies have applied the FOX-2 assay in PDT using porphyrins, a class of PSs that primarily undergo Type II mechanisms ​ (Melo et al. 2013). Phospholipid hydroperoxides were identified as the predominant photoproducts generated during photosensitization with cationic porphyrins. Moreover, the formation of HOO^•^ derivatives correlated with increased photosensitization efficiency in bacterial inactivation (Melo et al. 2013). Since phospholipids are key targets in photosensitization, their oxidation can serve as a valuable marker for assessing and monitoring photodamage in biological systems.

In PDT studies, the FOX-2 assay is typically performed in lipid vesicles (LUVs) containing specific concentrations of the PS, which are then exposed to radiation. The procedure generally follows a modified version of the method described by Wolff (Wolff 1994). The FOX-2 reagent is prepared by dissolving ferrous ammonium sulfate and sulfuric acid in water, followed by the addition of butylated hydroxytoluene (BHT), xylenol orange, and methanol to achieve the appropriate reaction conditions (Melo et al. 2013; Vignoni et al. 2018; Sosa et al. 2023).

The FOX-2 assay can be a valuable tool for assessing oxidative damage in PDT studies, particularly in evaluating lipid hydroperoxide formation. However, because ^1^O_2_- and radical-mediated peroxidation generate overlapping but non-identical hydroperoxide profiles, the assay cannot distinguish between these mechanisms without additional controls (Salyha and Oliynyk 2023b). Furthermore, under prolonged hypoxia, enhanced antioxidant defenses (elevated GSH, GPX4 activity) may reduce steady-state LOOH levels even when initial peroxidation rates are high, potentially leading to underestimation of oxidative damage if sampling is delayed. Time-course measurements and inclusion of GPX4 inhibitors (e.g., RSL3) as positive controls can help interpret FOX assay data in hypoxic models (Shui et al. 2021a; Hong et al. 2022).

#### TBARS assay

Lipid peroxidation is one of the most significant forms of oxidative damage, involving the degradation of phospholipids, cholesterol, and other unsaturated lipids. This process leads to the formation and breakdown of lipid hydroperoxides (LOOHs), generating stable and toxic oxidized compounds (Girotti 1990; Du et al. 2003) that compromise membrane integrity by reducing fluidity, increasing permeability, and decreasing membrane potential. Prolonged oxidative stress ultimately results in the destruction of cellular and organelle membranes, leading to cell death (Daicoviciu et al. 2011; Ion et al. 2012). Among the byproducts of lipid peroxidation, malondialdehyde (MDA) stands out as a toxic compound commonly used as a biomarker of oxidative stress (Saczko et al. 2004).

One widely used method for assessing lipid peroxidation is the thiobarbituric acid reactive substances (TBARS) assay, which detects MDA and other secondary peroxidation products. This test measures both the MDA naturally present in the sample and that generated from lipid hydroperoxides under assay conditions. Since MDA is a low-molecular-weight end product of lipid peroxidation, the TBARS assay provides an initial indication of oxidative damage and is particularly sensitive for detecting lipid peroxidation products such as 2,4-decadienals and, to a lesser extent, saturated aldehydes formed during the Type I mechanism (Ion et al. 2012). The reaction between thiobarbituric acid (TBA) and peroxidation products like MDA and 4-hydroxynonenal (HNE) results in TBARS, forming a pink-red trimethine adduct that can be quantified either by absorbance at 532 nm or fluorescence at 555 nm (Fig. 5G) (Girotti 1990; Babizhayev 1996). This reaction is conducted under high temperatures and acidic conditions to enhance sensitivity by promoting MDA adduct formation and accelerating fatty peroxide decomposition.

In a typical experiment, liposomes or cells are exposed to the PS at the desired concentration. After an incubation period of PS, the samples are submitted to irradiation at the appropriate wavelength for the PS used. At predetermined time points post-irradiation, the liposomes or cells are lysed using a lysis buffer containing an antioxidant such as BHT under controlled conditions. The samples are then centrifuged to remove insoluble material, and the supernatant is collected for analysis (Bacellar et al. 2014).

For MDA quantification, a calibration curve is prepared from a standard solution, diluted to obtain appropriate concentrations. To form the MDA-TBA adduct, a TBA solution is added to each standard or sample, followed by incubation at 95 °C for a fixed period. After cooling to room temperature, the absorbance or fluorescence of the MDA-TBA adduct is measured (Bacellar et al. 2014).

Bacellar et al*.* (2014) conducted a TBARS assay in a PDT study using liposomes prepared from soy lecithin films. The lipid films were hydrated to generate liposome suspensions, which were then treated with or without a PS and exposed to irradiation. Lipid peroxidation was evaluated by collecting samples at various time points postirradiation and analyzing the TBARS reaction products. Absorbance was measured at 532 nm, with background correction at 800 nm, and results were normalized to express relative levels of lipid oxidation across samples (Bacellar et al. 2014).

In PDT in vitro studies, MDA quantification can also be performed at different time points post-treatment to assess oxidative damage dynamics (Rapozzi et al. 2009; Karagöz et al. 2020; Shui et al. 2021b; de Melo Gomes et al. 2023; Kojima et al. 2024). Variations included the use of different TBA concentrations, incubation times, and wavelengths for detection, allowing adaptation to specific experimental conditions (Rapozzi et al. 2009; Karagöz et al. 2020; Shui et al. 2021b; de Melo Gomes et al. 2023; Kojima et al. 2024; Zhou et al. 2024). The data were typically expressed as MDA concentration relative to total protein or as absolute values in nmol/µL of TBARS. Despite methodological differences, all approaches aimed to assess MDA levels as an indicator of oxidative stress, with necessary controls and adjustments implemented to improve assay sensitivity and specificity. Under hypoxia, elevated TBARS levels relative to normoxic controls can thus serve as an indirect indicator of Type I pathway engagement, although the assay’s limited specificity means that other sources of reactive aldehydes must be controlled.

#### BODIPY-C11 (lipid peroxidation sensor)

Boron dipyrromethene (BODIPY) derivatives function as oxidation-sensitive probes for lipid peroxidation, undergoing a fluorescence shift from red to green upon oxidation (Cummins et al. 2013). These probes offer significant advantages, including intense and narrow fluorescence emission, as well as stability across a wide pH range and in various solvents (Cummins et al. 2013). Intracellular lipid peroxidation can be assessed using C11-BODIPY^581/591^ (Fig. 5H), with various methodological adaptations available to suit different cell types and implemented to ensure reliable detection across different experimental conditions.

In PDT studies, the oxidation-sensitive probe is typically added post-illumination to prevent direct photobleaching or photochemical reactions (Sakharov et al. 2005; Shui et al. 2021b; Ma et al. 2024; Xiong et al. 2024; Zhou et al. 2024). In fluorescence microplate assays (Rapozzi et al. 2009; dos Santos et al. 2020), cells are treated with C11-BODIPY and incubated under similar conditions, typically at a final concentration of 1 to 10 µM for 30 min at 37 °C after PDT treatment. Lipid peroxidation is quantified either by measuring the percentage decay of red fluorescence (non-oxidized form) in PDT samples compared to untreated controls (Rapozzi et al. 2009) or by assessing oxidized C11-BODIPY fluorescence at 488/510 nm (excitation/emission)—Fig. 5 (dos Santos et al. 2020). Fluorescence kinetics are monitored using microplate readers at 37 °C over a period of 1 h, with readings taken every 5 min to dynamically track oxidative changes. H_2_O_2_ treatment serves as a positive control for oxidative stress. Flow cytometry assays follow a similar protocol; C11-BODIPY is added post-PDT and probe incubation times can range from 30 min to 6 h at 37 °C. After C11-BODIPY incubation, cells are washed with PBS and re-suspended for flow cytometry analysis (Lange et al. 2019; Shui et al. 2021b; Zhang et al. 2023; Zhou et al. 2024).

Most C11-BODIPY-based lipid peroxidation assays employ fluorescence confocal microscopy. The probe is typically prepared in fetal calf serum at 20 µM and applied to cell cultures at a final concentration of 1 to 10 µM. Incubation lasts 10 to 30 min at 37 °C, either after PDT or, in some protocols, before irradiation (Zheng et al. 2022). Oxidized C11-BODIPY emits green fluorescence (excitation at 488 nm, emission at 500–530 nm), while the non-oxidized form emits red fluorescence (excitation at 543–561 nm, emission at 580–630 nm) (Sakharov et al. 2005; Rapozzi et al. 2009; Shui et al. 2021b; Ma et al. 2024; Xiong et al. 2024).

The BODIPY-C11 probe provides the advantages of real-time and spatially resolved detection of lipid peroxidation in live cells. This probe is particularly valuable for hypoxic PDT studies because it can capture the dynamics of peroxidation during and immediately after irradiation, before cellular repair mechanisms or secondary reactions obscure the initial oxidative burst (Shui et al. 2021a). However, because BODIPY-C11 is oxidized by both ^1^O_2_ and LOO^•^ radicals, it does not discriminate between Type I and Type II mechanisms without additional controls such as ^1^O_2_ quenchers (e.g., sodium azide) or radical scavengers (e.g., vitamin E analogs).

### Photoinduced oxidative damage to DNA by PSs

In PDT, PS-generated ROS can oxidize multiple classes of biomolecules, including lipids, proteins, and nucleic acids (Chen et al. 2021b; An et al. 2022; Moloudi et al. 2025). However, because ROS reactivity depends on the species generated, their intracellular localization, and the surrounding biochemical environment, the extent of DNA damage cannot be reliably inferred from total ROS production alone or from commonly reported endpoints such as mitochondrial depolarization, membrane disruption, or loss of cell viability (El-Hussein et al. 2012; Digby et al. 2019; Teng et al. 2025; El-Sadek et al. 2025). Lipids and proteins are generally the primary and most accessible targets of photooxidative reactions, whereas oxidative damage to nuclear DNA represents a more restricted subset of events (El-Hussein et al. 2012; Li et al. 2022b). When DNA is reached by ROS, PDT can induce oxidative base lesions, single- and double-strand breaks, DNA cross-linking, and other forms of genomic damage, which can be detected using comet assays and lesion-specific enzymes such as FPG and Endonuclease III El-Hussein et al. 2012). Indeed, DNA photodamage depends not only on ROS abundance and identity but also on ROS proximity to the nucleus, chromatin accessibility, nucleotide reactivity, and the efficiency of DNA repair mechanisms (El-Hussein et al. 2012; Digby et al. 2019; Moloudi et al. 2025).

Among the mechanisms involved in DNA oxidation during PDT, Type I photochemistry is considered a major contributor. Electron-transfer reactions generate radical species that can ultimately produce highly reactive oxidants, including ^•^OH, which are recognized as major mediators of oxidative damage to DNA bases and the sugar-phosphate backbone under oxidative stress conditions (El-Hussein et al. 2012; Lv et al. 2018; Teng et al. 2025; Moloudi et al. 2025). Consequently, although several ROS may contribute to DNA oxidation, ^•^OH is considered one of the most relevant candidates for inducing strand breaks and oxidative base lesions during Type I PDT (Lv et al. 2018; Li et al. 2020; An et al. 2022; Teng et al. 2025).

The extent of DNA photodamage is strongly influenced by the subcellular localization and DNA-binding properties of the PS. For example, the DNA-binding PS Br-DAPI induced extensive double-strand breaks only when physically associated with DNA (Digby et al. 2019). Mitochondrial DNA may be particularly susceptible because mitochondria are major sites of ROS generation and mtDNA lacks the protection provided by histones, while mitochondrial damage can additionally promote membrane permeabilization, cytochrome *c* release, caspase activation, and secondary nuclear DNA fragmentation (Moloudi et al. 2025).

This issue is especially relevant under hypoxia, where the suppression of ^1^O_2_ production can limit conventional Type II PDT, but Type I PSs may preserve phototoxicity through electron-transfer reactions and the generation of O_2_^−•^ and ^•^OH (Teng et al. 2025; El-Sadek et al. 2025). For instance, the cyclometalated Ru(II) PS Ru2 maintained PDT efficacy under both normoxic and hypoxic conditions, with ^•^OH formation identified as an important contributor to cell death (Lv et al. 2018). Similarly, oxygen-economizing systems such as SORgenTAM can generate O_2_^−•^, promote its conversion into ^•^OH through intracellular catalytic cascades, and reduce the oxygen dependence of PDT (Li et al. 2020). Nevertheless, persistent ROS production under hypoxia does not necessarily demonstrate that DNA is the primary intracellular target of photodamage (El-Hussein et al. 2012; Digby et al. 2019; An et al. 2022). Therefore, the evaluation of photoinduced DNA oxidation should rely on direct evidence, including strand-break assays (David et al. 2005; El-Hussein et al. 2012), DNA damage signaling markers (Moloudi et al. 2025), oxidative base modification assays (El-Hussein et al. 2012), and experimental confirmation that the PS localizes to, or exerts its photochemical activity near, nuclear DNA (Digby et al. 2019). Under hypoxic conditions, such analyses are particularly important because DNA damage may arise not only from residual ^1^O_2_ production but also from Type I radical chemistry, subcellular localization of the PS, mitochondrial dysfunction, and activation of the cellular DNA damage response.

## Conclusions and future perspectives

PDT remains a powerful and versatile therapeutic modality; however, its biological efficacy is fundamentally constrained by oxygen availability and by the experimental paradigms used to evaluate PSs. As critically discussed in this review, the widespread reliance on normoxic in vitro models has led to a systematic overestimation of PDT performance and an implicit bias toward Type II, ^1^O_2_–dominated photochemistry. Under hypoxic conditions, which more closely reflect the tumor microenvironment even at the cellular scale, PDT efficacy is reshaped by coordinated changes in PS uptake, subcellular localization, ROS profiles, and biomolecular damage patterns.

A central conclusion emerging from this analysis is that hypoxia-driven resistance to PDT does not arise solely from oxygen depletion, but from broader cellular reprogramming involving metabolism, membrane organization, and stress-response pathways. These adaptations alter the intracellular location of PSs and may impact the dominant photochemical mechanisms from oxygen-dependent Type II pathways toward radical-based Type I processes. This mechanistic shift has important implications for how oxidative damage to biomolecules is generated and detected. Under hypoxia, lipid oxidation may proceed through radical-mediated, while protein oxidation emerges as a persistent and functionally relevant target of Type I photochemistry. Many commonly used analytical tools—including ROS probes and assays for lipid and protein oxidation—were developed and validated under normoxic conditions and therefore require careful adaptation and interpretation to capture hypoxia-dominated PDT mechanisms and to accurately assess PDT outcomes under low oxygen conditions.

Looking forward, advancing PDT toward hypoxia-relevant applications requires both conceptual and methodological realignment. From a PS design perspective, greater emphasis should be placed on molecules capable of sustaining photodynamic activity under low oxygen concentration, including those favoring Type I pathways, targeting organelles outside the most oxygen-depleted compartments, or combining hybrid Type I/Type II reactivity. Importantly, intrinsic photophysical properties must also be evaluated alongside PS behavior in hypoxia-adapted cellular environments, rather than inferred from solution-based measurements alone.

Equally critical is the refinement of in vitro experimental models. Controlled oxygen culture systems, hypoxia-adapted cell lines, and three-dimensional (3D) spheroids should be incorporated into PDT evaluation pipelines to capture oxygen gradients, metabolic heterogeneity, and diffusion constraints absent in conventional monolayers. Comparative analyses across normoxic and hypoxic conditions, as well as between 2 and 3D architectures, are essential to disentangle intrinsic PS properties from context-dependent cellular responses.

In summary, overcoming hypoxia-driven resistance in PDT is not simply a matter of improving oxygen delivery or maximizing Type I-PS, but of embracing the complexity of cellular adaptation and photochemical diversity under low oxygen conditions. By redefining how PDT is modeled, measured, and interpreted in vitro, the field can move toward more mechanistically sound and biologically relevant strategies for developing next-generation PSs capable of operating effectively in hypoxic environments.

## Data Availability

No datasets were generated or analyzed during the current study.
